# Costs and Outcomes of a Therapist-Guided Internet-Delivered Cognitive Behavioral Therapy: Multicenter Observational Study

**DOI:** 10.2196/73067

**Published:** 2025-07-28

**Authors:** Zareen Abbas Khan, Jørn Heggelund, Stian Lydersen, Kristian Kidholm, Henrik Pedersen, Imre Janszky, Tine Nordgreen, Vidar Halsteinli

**Affiliations:** 1 Research and Development Unit Department of Mental Health Care St Olav's University Hospital Trondheim Norway; 2 Department of Public Health and Nursing Faculty of Medicine and Health Sciences Norwegian University of Science and Technology Trondheim Norway; 3 Department of Mental Health Faculty of Medicine and Health Sciences Norwegian University of Science and Technology Trondheim Norway; 4 Department of Mental Health Regional Centre for Child and Youth Mental Health and Child Welfare Norwegian University of Science and Technology Trondheim Norway; 5 Center for Innovative Medical Technology University of Southern Denmark Odense Denmark; 6 Nidaros Community Mental Health Centre Division of Psychiatry St Olav's University Hospital Trondheim Norway; 7 Division of Psychiatry Haukeland University Hospital Bergen Norway; 8 Department of Global Public Health and Primary Care University of Bergen Bergen Norway

**Keywords:** telemedicine, MeSH, Medical Subject Headings, depression, social anxiety, panic disorder, health service delivery, routine care, specialist mental health care

## Abstract

**Background:**

Therapist-guided internet-delivered cognitive behavioral therapy (iCBT) has demonstrated efficacy and potential cost-effectiveness in treating depression, anxiety, and panic disorder in randomized controlled trials (RCTs). However, evidence of its outcomes and costs in routine care settings on a national level remains limited.

**Objective:**

We aimed to assess the program costs and outcomes of therapist-guided iCBT for depression, social anxiety, and panic disorder posttreatment (T2) and at 6-month follow-up (T3) in a broad context, in addition to exploring how the program’s costs and effects vary by hospital.

**Methods:**

This single-arm observational study analyzed patient-reported data collected from routine care between 2021 and 2024 in 4 hospitals in Norway. The symptom severity of depression, social anxiety, and panic disorder was measured using the 9-item Patient Health Questionnaire (PHQ-9), the Social Phobia Inventory (SPIN), and the Panic Disorder Severity Scale (PDSS), respectively. Generic health-related quality of life (HRQoL) was measured with the EQ-5D-5L, while work and social functioning were measured with the Work and Social Adjustment Scale (WSAS) and sick leave days. Mixed effects models estimated changes in outcomes over time and between hospitals. Hospital-specific annual program costs per patient were estimated based on infrastructure and therapist guidance expenses. The economic evaluation was performed with a hospital perspective and extended to a societal perspective by examining sick leave days.

**Results:**

Data from 565 participants showed substantial improvements across all outcomes at T2 and T3. At T2, 102 (35.1%) participants responded positively to treatment (depression: 44/140, 31.4%; social anxiety: 21/79, 26.6%; panic disorder: 37/72, 51.4%), and 66 (23.7%) achieved remission (depression: 17/139, 12.2%; social anxiety: 26/73, 35.6%; panic disorder: 23/67, 34.3%). Regarding work and social functioning, 97 (33.0%) patients responded positively to the treatment and 60 (23.0%) achieved remission, as measured by the WSAS. Patients reported a mean reduction of 3.2 sick leave days (95% CI –5.4 to –0.9) at T2 and 7.7 sick leave days (95% CI –11.5 to –3.9) at T3. EQ-5D-5L utility scores increased by a mean of 0.11 (95% CI 0.08-0.13) at T2 and 0.12 (95% CI 0.09-0.15) at T3. Patient-reported outcomes were consistent across time points and hospitals and robust to sensitivity analyses accounting for patient and hospital characteristics and missing data scenarios. The mean total program costs per patient were US $1030.12 (SD 451.6), which varied by location (US $636.41-$2152.47), mostly due to differences in patient volume.

**Conclusions:**

This study confirms the potential of therapist-guided iCBT to relieve symptom severity and improve well-being across different health service providers when implemented as part of routine specialist health care. The observed variance in costs per patient between hospitals underscores the importance of patient volume to optimize efficient use of resources.

## Introduction

### Background

Internet-delivered cognitive behavioral therapy (iCBT) is a well-established treatment for several mental health disorders [1-3]. Its efficacy in depression [4,5], social anxiety [4,6,7], and panic disorder [8] has been demonstrated in numerous clinical trials compared to treatment as usual [5], face-to-face therapy [4,8,9], and inactive controls [7]. There is mixed evidence regarding its cost-effectiveness compared to standard care (eg, face-to-face therapy [10,11]), but it is likely cost-effective compared to inactive controls, such as waiting lists or psychological placebos [12]. Patients have reported a high level of satisfaction with iCBT [13], and for certain patient groups, digital therapy may increase the ease of access to care [14,15]. Given these advantages, iCBT has been successfully implemented in many countries [16] and continues to gain traction as a scalable solution for mental health care [15].

Despite these promising developments, an ongoing challenge is determining whether the robust outcomes observed in controlled trials are replicable in real-world settings [17]. Trials often involve controlled environments and static protocols, which may not reflect the variability and challenges encountered in routine practice [18]. Randomized controlled trials (RCTs) often exclude patients based on age, gender, or comorbidities, which can compromise the generalizability of results in clinical practice [19]. Moreover, real-world effectiveness may be influenced by a range of factors, including organizational differences [20,21], therapists’ attitudes [17,22,23], patients’ perceptions [24,25], and the ease of using the technology [26]. To the best of our knowledge, no study has yet examined how costs and outcomes of the same iCBT treatment vary across different locations when implemented on a large scale in routine specialist mental health care.

Prioritization and decision-making in health care often rely on single metrics, such as the incremental cost-effectiveness ratio (ICER) [27]. However, such metrics may oversimplify the nuanced costs and benefits of complex digital health interventions. For example, Jankovic et al [28] underscore that the cost-effectiveness of digital health interventions may vary depending on the analysis viewpoint, the choice of comparator, and the implementation setting. Similarly, Gomes et al [29] and McNamee et al [30] argue that a wider set of outcomes, such as nonhealth benefits (eg, patient satisfaction, accessibility), as well as health system efficiency, should be considered for complex digital health interventions. Among the several proposed frameworks, the Model for Assessment of Telemedicine Applications (MAST) offers a multidisciplinary assessment spanning from application characteristics and patient perspectives to economic and organizational aspects that extend beyond the traditional cost-effectiveness approach within health technology assessment [31].

The nationwide implementation of eCoping (eMeistring) [32,33] in Norway provides a unique opportunity to explore the real-world performance of guided iCBT in routine specialist care [34]. It also allows for the assessment of outcomes in a broader context and the evaluation of annual cost variations across hospitals. The comparative effectiveness of eCoping, and similar guided iCBT programs, has been examined in previous trials [35-39].

### Objectives

This study aimed to evaluate patients’ self-reported outcomes posttreatment (T2) and at 6-month follow-up (T3) after a routine care therapist-guided iCBT program for depression, social anxiety, and panic disorder. Outcomes included symptom severity, work and social functionality, and health-related quality of life (HRQoL). Additionally, we investigated whether patients’ outcomes and program costs per patient differ between hospitals.

## Methods

### Study Design and Inclusion of Patients

The study was designed as a single-arm multicenter observational trial at 4 hospitals (Haukeland University Hospital, H1; St Olavs Hospital, H2; Vestfold Hospital, H3; and Innlandet Hospital, H4). Since the trial did not have a comparator group, a cost-outcome descriptive analysis was undertaken [27]. We took a hospital perspective in estimating the program costs and extended them to a societal perspective by examining sick leave days.

Patients receiving eCoping at the participating hospitals were invited. The study started in September 2021 with H1 and H2, whereas H3 and H4 were included in 2022 and 2023, respectively. The study ended on June 30, 2024, for all 4 hospitals. All patients completed questionnaires at 3 time points, baseline (T1), T2, and T3, as part of the routine care pathway. They received automatic reminders for questionnaire completion.

eCoping was offered to patients (aged 18 years or older) if they had a diagnosis of major depressive disorder, social anxiety disorder, or panic disorder; met the national criteria on the need for specialized treatment; and were proficient in reading and writing Norwegian. Patients were offered alternative treatments within specialist mental health care if they were experiencing acute suicidal ideation, psychosis, or substance abuse; lacked internet access; or displayed a need or preference for another treatment modality. Eligibility was determined through a face-to-face clinical assessment [32].

### Ethical Considerations

This study was approved by the Regional Committee for Medical and Health Research Ethics in Norway (2015/878). All procedures contributing to this study adhered to the ethical standards established by the Norwegian Regional Ethics Committee (REK), as well as the principles outlined in the Declaration of Helsinki.

All included participants provided informed consent in accordance with Norwegian law and recommendations. They did not receive any compensation and were free to withdraw consent at any time. Patients who consented to participate were included in the analysis if they answered at least 1 outcome-related questionnaire at T1. Therapists were blinded to patients’ study participation.

### Treatment

eCoping is a therapist-guided standardized iCBT program for depression, social anxiety, and panic disorder. The program’s content and duration have been described in detail elsewhere [32,33,39]. It was accepted for national implementation into specialist health services in Norway in 2019 based on a health technology assessment provided by the Norwegian Institute of Public Health [40]. The program has demonstrated clinical effectiveness in previous trials [32,33,39] and is currently available through most health regions in Norway.

Therapists involved in the program are licensed health care professionals, including clinical psychologists, registered nurses, and clinical social workers, who underwent training and were supervised by a specialist psychologist. The therapists provided 10-15 minutes of weekly feedback to patients through the eCoping platform and followed up via secure messages, texts, or calls, as needed. Patients at risk of adverse events were followed up according to the existing guidelines for specialist mental health services in Norway.

### Participating Hospitals

All 4 hospitals are general public hospitals serving a specific geographical area with specialized somatic and mental health services: H1 (364,000 inhabitants), H2 (269,000 inhabitants), H3 (201,000 inhabitants), and H4 (276,000 inhabitants). At each hospital, outpatient units organized within community mental health centers were responsible for iCBT delivery. H1 initiated the eCoping program in Norway in 2014, shortly followed by H3 in the same year. H2 began eCoping in 2018 and H4 in 2021. Patients were referred by general practitioners (GPs) for all hospitals (“GP-referred”). In addition, H1 and H2—both large university hospitals—allowed patients to contact the hospitals directly (“self-referred”). At H1, patients self-referred via a secure message through a public health website, while H2 accepted self-referrals via email to the outpatient clinic. The treatment information is available on each hospital’s website. Outreach to patients was done via social media and newspapers, whereas GPs were informed about the program by eCoping staff.

### Outcomes

Patients’ symptom severity, functional impairment, sickness absenteeism, and the HRQoL were measured using self-administered questionnaires on the eCoping technical platform provided by the company Checkware AS [41]. The following questionnaires were included in the analysis:

- The 9-item Patient Health Questionnaire (PHQ-9) [42]: The PHQ-9 is a 9-item instrument that measures depression severity on a scale of 0 (“not at all”) to 3 (“nearly every day”) based on the major depression criteria of the *Diagnostic and Statistical Manual of Mental Disorders, Fifth Edition* (*DSM-5*). Total scores range from 0 to 27, with higher scores indicating more severe depression. The scale has been shown to be reliable and responsive to change in other samples [43,44]. Internal consistency in our sample yielded Cronbach α=.85 at T1. Outcomes were reported as changes in mean scores, response (≥50% reduction from T1) [45], and remission (PHQ-9 score≤5) [46].
- The Social Phobia Inventory (SPIN) [47]: SPIN measures the severity of social anxiety through 17 items, each rated on a 5-point scale, ranging from 0 (“none”) to 4 (“extreme”). Total scores range from 0 to 68, with scores above 19 indicating a need for further clinical evaluation. In our sample, T1 Cronbach α=.89, indicating high internal consistency. Outcomes were reported as mean score changes, response (≥55% reduction from T1) [47], and remission (SPIN score≤19).
- The Panic Disorder Severity Scale (PDSS) [48]: The PDSS measures the severity of panic disorder through 7 items, each rated on a 5-point scale, ranging from 0 (“none”) to 4 (“extreme”). Total scores range from 0 to 28, with scores above 9 indicating a need for a formal diagnostic assessment. In our sample, T1 Cronbach α=.87, indicating good internal consistency. Outcomes were reported as mean score changes, response (≥40% reduction from T1) [48], and remission (PDSS score≤5).
- The 7-item Generalized Anxiety Disorder scale (GAD-7) [49]: The GAD-7 assesses anxiety severity through 7 items, each rated from 0 (“not at all”) to 3 (“nearly every day”). Total scores range from 0 to 21, with higher scores indicating more severe anxiety. The GAD-7 has demonstrated strong psychometric properties and sensitivity in detecting anxiety disorders in previous studies [50]. In our sample, T1 Cronbach α=.86, indicating good internal consistency. Outcomes were reported as mean score changes by hospital in the main analysis and by time in the supplementary analysis.
- The Work and Social Adjustment Scale (WSAS) [51]: The WSAS is a 5-item questionnaire assessing the impact of mental health difficulties on daily functioning, including work, leisure, social activities, and personal and family relationships. Scores range from 0 to 40, with a score above 20 indicating severe impairment. The WSAS has demonstrated high internal consistency in previous studies [51], and in our sample, Cronbach α=.77 at T1. Outcomes were reported as mean score changes, response (≥8 point reduction from T1) [52], and remission (WSAS score≤10) [51].
- The EQ-5D-5L [53]: The EQ-5D-5L is a generic HRQoL measure assessing 5 dimensions of health. Each dimension is rated on a Likert scale from 1 to 5, with responses converted to a health state index value using a preference-based weight. This typically ranges from 0 to 1, where 1 indicates perfect health, while 0 indicates a health state equivalent of death. States below 0 are also possible, indicating health-related quality worse than death. Outcomes were reported as differences in the mean index values using Norwegian preference weights for all time points [54].
- Sickness-related absence: Patients reported the number of days they were absent from work in the past 4 weeks. This outcome was reported as the mean change in days absent.

### Costs

Program costs per patient were estimated separately for each hospital following the published checklist by Khan et al [55]. Program costs were categorized as follows:

- Infrastructure costs: These comprised maintenance and implementation costs. Maintenance costs included hosting, server storage, data management, and fees to the company providing the technical platform for iCBT treatment. Implementation costs were based on the time associated with training clinicians for the eCoping treatment and costs related to program administration and local management. Detailed cost information was collected through close contact with administrative staff at each of the participating hospitals. Infrastructure costs were defined as a fixed yearly cost per patient. The share of annual total iCBT infrastructure cost belonging to study participants was calculated and subsequently divided by the number of patients in the study. For patients starting treatment in 2022 or before, infrastructure costs for 2022 were used, whereas 2023 costs were applied for all other patients.
- Therapists’ costs: The cost of therapist guidance was based on hospital-specific mean time estimates for the different digital (and in-person) therapist-patient contacts as part of the standardized program. A therapist unit price per hour was calculated for each hospital according to the skill mix of therapists (ie, clinical psychologists, nurses, and social workers) and hospital wage levels. Separate unit costs for 2022 and 2023 were estimated. Data on average therapists’ time per contact, skill mix, and wage levels were obtained from contact with clinicians, administrative staff, quarterly reports, and questionnaires. Thereafter, the therapist cost per patient was calculated by multiplying each patient’s aggregated therapist time (hours) with the therapist unit price. Therapist costs varied between patients according to the number of modules completed. For patients starting treatment in 2022 or before, the therapist unit price for 2022 was used, whereas 2023 costs were applied for all other patients.

The total program cost for each patient was estimated by adding the fixed infrastructure cost and the variable therapist cost. We adjusted 2023 infrastructure costs and the therapist unit price per hour to 2022 using the inflation index from Statistics Norway [56]. Norwegian kroner (NOK) were converted to US dollars (US $) using the conversion rate of US $1=NOK 9.62 in 2022 [57].

### Statistical Analyses

Descriptive statistics were presented as means (SDs) for continuous variables and as counts and percentages for categorical variables. We report 95% CIs, where relevant, and considered 2-sided *P*<.05 to indicate statistical significance. All analyses were conducted using Stata SE version 18.5[58].

In the main analysis, we analyzed symptom-related instruments separately by treatment group (PHQ-9 for depression, SPIN for social anxiety disorder, and PDSS for panic disorder), while work and social impairment (WSAS), EQ-5D-5L, and sickness-related absence were analyzed collectively. We estimated treatment effects over time (ie, at T2 and T3) using linear mixed effects models. Each patient-reported outcome was analyzed adjusting for hospital (H1-H4), age, and gender as fixed effects. “Patient” was treated as a random effect. Normality of residuals for the linear models was assessed through visual inspection of quantile-quantile plots. Mixed models provide unbiased estimates under the assumption that data are missing at random (MAR), while a complete case analysis would be unbiased only under the more restrictive missing completely at random (MCAR) assumption. The results from these models were reported as regression coefficients for the effect of time. Additionally, we estimated standardized effect sizes for the PHQ-9, SPIN, the PDSS, and the WSAS using Cohen d, which is calculated as the adjusted mean difference from the mixed model divided by the SD of the variable at T1. The main analyses were conducted following the intention-to-treat (ITT) principle.

The hospital-level effect was assessed by including the interaction between hospital and time in mixed effects models. To maximize statistical power, hospital-time interaction analysis was conducted for instruments where all participants provided data regardless of treatment assignment (ie, PHQ-9, GAD-7, WSAS, and EQ-5D-5L). The predicted scores for each hospital with respect to time were examined via line plots. To statistically test the significance of the hospital-level effect, a likelihood ratio (LR) test was performed comparing the time-hospital interaction model with “hospital” as a fixed covariate only. The resulting *P* values from the LR test were reported alongside the line plots.

To test the robustness of our findings, several sensitivity analyses were conducted. Patient-reported outcomes were examined over time across a broader set of measures within each treatment group. The model was adjusted for additional covariates (ie, referral pathway, employment status, and medication use) to assess whether these factors alter the fixed effect of time or hospitals on treatment outcomes. Treatment completion was explored by restricting the analysis to participants who completed at least 4 of 8 modules in the depression group and 5 of 9 modules in the social anxiety and panic disorder groups. To investigate differences in outcomes between hospitals, additional analyses were conducted based on hospital characteristics. H1 was compared with all other hospitals, as it was the first to implement eCoping and has the most experience with the treatment. A second comparison grouped H1 and H2, both large university hospitals with self-referral pathways, against H3 and H4, which are smaller and accept patients by GP referral only. Finally, following the recommendations by Faria et al [59] and Haque et al [60], the potential impact of data missing not at random was assessed by imputing missing data using chained equations (MICE), followed by delta adjustment of 10%-30%, assuming worse outcomes among those with missing data. The relevant Stata code is available in Multimedia Appendix 1.

## Results

### Participants’ Characteristics at T1

**Figure 1:**
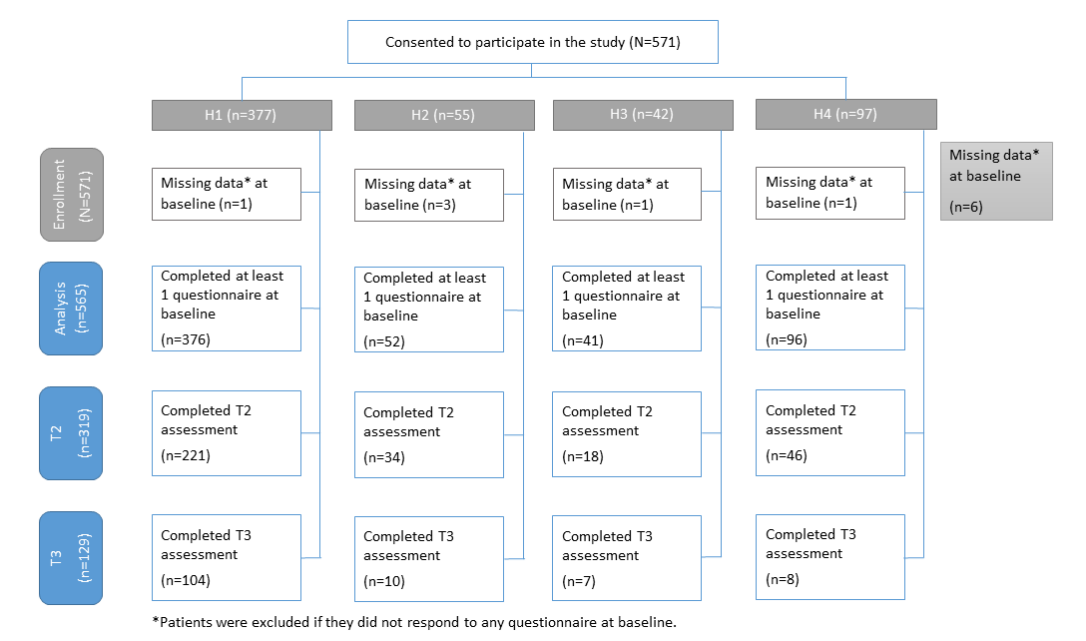
Study flowchart. T2: posttreatment; T3: 6-month follow-up.

A detailed description of the study sample, including patients who provided consent and met the inclusion criteria, is presented in the flowchart in Figure 1. The characteristics of the participating patients at T1 are summarized in Table 1. A total of 1150 patients were invited to participate in the study, of which 571 (49.7%) consented to participate (Figure 1). The majority received treatment in H1 (376/565, 66.5%), followed by H4 (96/565, 17.0%), H2 (52/565, 9.2%), and H3 (41/565, 7.3%). None of the patients in the social anxiety group at H3 consented to participate. At H3 and H4, education and civil status were not part of the routine care T1 data collection due to the local data protection. Of the study’s 565 participants, 356 (63.0%) were females, 226 (40.0%) were single or living alone, and the mean age was 32.9 (SD 10.9) years. The proportion with higher education was 41.7% (177/424), while 76.4% (425/556) were either employed or students. Participants had moderately high symptom severity and impairment, as indicated by their PHQ-9 (mean 14.0, SD 5.74), SPIN (mean 40.3, SD 11.87), PDSS (mean 12.3, SD 5.52), and WSAS (mean 21.0, SD 8.25) scores. The mean EQ-5D-5L utility score at T1 was 0.59 (SD 0.25). In total, 151 (26.7%) of the patients were self-referred, whereas 413 (73.1%) were referred by either a GP or a specialist. A total of 169 (30.3%) patients were taking antidepressants or anxiolytics at T1, with H3 (24/41, 58.5%) and H4 (55/96, 57.3%) reporting a higher proportion of medicine use in the sample compared to H1 (137/376, 36.4%) and H2 (20/52, 38.5%).

**Table 1:** Characteristics of participants at T1^a^ (overall and by hospital).

Characteristics		Total participants (N=565)	H1^b^ (n=376)	H2^c^ (n=52)	H3^d^ (n=41)	H4^e^ (n=96)
**Gender, n (%)**						
	Female	356 (63.0)	246 (65.4)	26 (50.0)	21 (51.2)	63 (65.6)
	Male	209 (37.0)	130 (34.6)	26 (50.0)	20 (48.8)	33 (34.4)
**Education** ^f^ **, n (%)**						
	Primary	41 (7.3)	38 (10.1)	3 (5.8)	N/A^g^	N/A
	Secondary	179 (31.7)	152 (40.4)	27 (51.9)	N/A	N/A
	Higher	177 (31.3)	158 (42.0)	19 (36.5)	N/A	N/A
	Missing	168 (29.7)	28 (7.4)	3 (5.8)	41 (100)	96 (100)
**Civil status^f^, n (%)**						
	Single	226 (40.0)	191 (50.8)	35 (67.3)	N/A	N/A
	Married/cohabiting	198 (35.0)	182 (48.4)	16 (30.8)	N/A	N/A
	Missing	141 (25.0)	3 (0.8)	1 (1.9)	41 (100)	96 (100)
**Referral type, n (%)**						
	Self-referred	151 (26.7)	139 (37.0)	12 (23.1)	0	0
	GP^h^-referred	413 (73.1)	236 (62.8)	40 (76.9)	40 (97.6)	96 (100)
	Missing	1 (0.4)	1 (0.3)	0	1 (2.4)	0
**Treatment group, n (%)**						
	Depression	271 (48.0)	176 (46.8)	22 (42.3)	32 (78.0)	41 (42.7)
	Social anxiety	157 (27.8)	103 (27.4)	19 (36.5)	0	35 (36.5)
	Panic disorder	137 (24.2)	97 (25.8)	11 (21.2)	9 (22.0)	20 (20.8)
**Medicine use, n (%)**						
	Antidepressants	73 (12.9)	44 (11.7)	2 (3.8)	5 (12.2)	22 (22.9)
	Anxiolytics	96 (17.0)	59 (15.7)	11 (21.2)	8 (19.5)	18 (18.8)
	Hypnotics	20 (3.5)	8 (2.1)	1 (1.9)	4 (9.8)	7 (7.3)
	Pain medication	7 (1.2)	5 (1.3)	0	2 (4.9)	0
	Psychostimulants	10 (1.8)	2 (0.5)	2 (3.8)	1 (2.4)	5 (5.2)
	Other	30 (5.3)	19 (5.1)	4 (7.7)	4 (9.8)	3 (3.1)
	None	320 (56.6)	234 (62.2)	30 (57.7)	17 (41.5)	39 (40.6)
	Missing	9 (1.6)	5 (1.3)	2 (3.8)	0	2 (2.1)
**Employment status, n (%)**						
	Employed	377 (66.7)	267 (71.0)	24 (46.2)	28 (68.3)	58 (60.4)
	Student	48 (8.5)	21 (5.6)	14 (26.9)	2 (4.9)	11 (11.5)
	Welfare benefits^i^	101 (17.9)	64 (17.0)	10 (19.2)	6 (14.6)	21 (21.9)
	Other^j^	30 (5.3)	20 (5.3)	2 (3.8)	5 (12.2)	3 (3.1)
	Sickness-related absence^k^	210 (37.2)	142 (37.8)	11 (21.2)	19 (46.3)	38 (39.6)
	Missing	9 (1.6)	4 (1.1)	2 (3.8)	0	3 (3.1)
Age (years; n=563), mean (SD)		32.9 (10.9)	33.3 (10.9)	29.4 (10.1)	34.7 (11.9)	32.3 (10.8)
Symptom duration (years; n=156)^f^, mean (SD)		7.9 (9.9)	8.0 (10.1)	7.2 (8.2)	N/A	N/A
**Workweek (n=371), mean (SD)**						
	Hours worked	33.2 (16.2)	33.5 (13.2)	33.9 (31.0)	33.0 (20.9)	30.5 (13.8)
	Days worked	4.5 (1.4)	4.5 (1.4)	4.3 (1.4)	4.1 (1.3)	4.5 (1.6)
**Symptom severity and functioning, mean (SD)**						
	PHQ-9^l^ (n=558)	14.0 (5.7)	13.7 (5.8)	13.4 (6.2)	15.1 (5.5)	14.8 (5.4)
	GAD-7^m^ (n=557)	10.7 (4.8)	10.4 (4.9)	9.9 (4.1)	11.9 (4.8)	11.8 (4.6)
	SPIN^n^ (n=155)	40.3 (11.9)	40.1 (11.9)	38.0 (15.2)	N/A	42.0 (9.8)
	PDSS^o^ (n=136)	12.3 (5.5)	12.5 (5.5)	10.4 (3.9)	12.1 (4.8)	12.7 (6.5)
	WSAS^p^ (n=559)	21.0 (8.2)	20.5 (8.3)	20.8 (8.4)	22.5 (8.0)	22.3 (7.9)
	EQ-5D-5L (n=559)	0.59 (0.25)	0.60 (0.26)	0.63 (0.21)	0.56 (0.26)	0.55 (0.24)

^a^T1: baseline.

^b^H1: Haukeland University Hospital.

^c^H2: St Olavs Hospital.

^d^H3: Vestfold Hospital.

^e^H4: Innlandet Hospital.

^f^Based on information from H1 and H2 only.

^g^N/A: not applicable.

^h^GP: general practitioner.

^i^Long-term sickness, disability, job seeker, and job training.

^j^Homemaker, retired, or other.

^k^Any sickness-related absence in the past 4 weeks for those who are employed.

^l^PHQ-9: 9-item Patient Health Questionnaire.

^m^GAD-7: 7-item Generalized Anxiety Disorder scale.

^n^SPIN: Social Phobia Inventory.

^o^PDSS: Panic Disorder Severity Scale.

^p^WSAS: Work and Social Adjustment Scale.

### Patient-Reported Outcomes Over Time

Table 2 presents descriptive statistics and results from the mixed models, including standardized effect sizes for each outcome. The models show that patients had a reduction in symptom severity, as measured by the PHQ-9, SPIN, and the PDSS at T2, with improvements maintained at T3. For example, for the depression program, a mean reduction of –4.4 and –5.5 points on the PHQ-9 was found at T2 and T3, with effect sizes of –0.85 and –1.08, respectively. Additionally, when analyzing the total sample, the WSAS score reduced at both T2 and T3, with effect sizes of –0.59 and –0.85, respectively. The EQ-5D-5L utility score improved at T2 and was maintained at T3, increasing by 0.11 and 0.12 points, respectively, from T1 (0.59). Furthermore, patients reported a reduction of 3.2 sick leave days at T2 and 7.7 sick leave days at T3 compared to T1. Regression coefficients, indicating the fixed effect of time, were statistically significant for all patient-reported outcomes at both T2 and T3. Sensitivity analyses indicated that the regression coefficients for the fixed effect of time were robust to adjustment of additional covariates and did not vary greatly for those who fulfilled the predefined criteria for treatment completion (Tables S1-S3 in Multimedia Appendix 1).

**Table 2:** Observed and predicted patient-reported outcomes over time.

Group/outcome			Descriptive statistics						Regression coefficient for time		
			Time point		Participants, n (%)		Mean (SD)		Estimate (95% CI)^a^		Standardized effect size^b^
**Depression**											
	PHQ-9^c^	T1^d^		267 (98.5)		15.9 (5.1)		N/A^e^		N/A	
	PHQ-9	T2^f^		143 (52.8)		11.1 (6.0)		–4.4 (–5.2 to –3.6)		–0.85	
	PHQ-9	T3^g^		56 (20.7)		9.1 (5.4)		–5.5 (–6.7 to –4.3)		–1.08	
**Social anxiety disorder**											
	SPIN^h^	T1		155 (98.7)		40.3 (11.9)		N/A		N/A	
	SPIN	T2		81 (51.6)		26.6 (15.4)		–13.2 (–16.0 to –10.4)		–1.11	
	SPIN	T3		28 (17.8)		22.4 (15.9)		–15.5 (–19.9 to –11.1)		–1.30	
**Panic disorder**											
	PDSS^i^	T1		136 (99.3)		12.3 (5.5)		N/A		N/A	
	PDSS	T2		73 (53.3)		7.8 (5.3)		–4.4 (–5.5 to –3.3)		–0.79	
	PDSS	T3		42 (30.7)		6.5 (5.0)		–5.3 (–6.7 to –4.0)		–0.96	
**All**											
	WSAS^j^	T1		559 (98.9)		21.0 (7.8)		N/A		N/A	
	WSAS	T2		299 (52.9)		15.8 (9.1)		–4.6 (–5.4 to –3.8)		–0.59	
	WSAS	T3		126 (22.3)		12.2 (8.0)		–6.6 (–7.9 to –5.5)		–0.85	
	EQ-5D-5L	T1		554 (98.1)		0.59 (0.24)		N/A		N/A	
	EQ-5D-5L	T2		308 (54.5)		0.72 (0.21)		0.11 (0.08 to 0.13)		N/A	
	EQ-5D-5L	T3		129 (22.8)		0.78 (0.18)		0.12 (0.09 to 0.15)		N/A	
	Sick leave (days)	T1		231 (61.3)		15.5 (11.8)		N/A		N/A	
	Sick leave (days)	T2		122 (32.4)		12.5 (12.2)		–3.2 (–5.4 to –0.9)		N/A	
	Sick leave (days)	T3		40 (10.6)		9.9 (10.3)		–7.7 (–11.5 to –3.9)		N/A	

^a^Fixed effect of time, adjusted for age, gender, and hospital as covariates.

^b^Standardized effect size (Cohen d).

^c^PHQ-9: 9-item Patient Health Questionnaire.

^d^T1: baseline.

^e^N/A: not applicable.

^f^T2: posttreatment

^g^T3: 6-month follow-up.

^h^SPIN: Social Phobia Inventory.

^i^PDSS: Panic Disorder Severity Scale.

^j^WSAS: Work and Social Adjustment Scale.

### Patient-Reported Outcomes by Hospital

**Figure 2:**
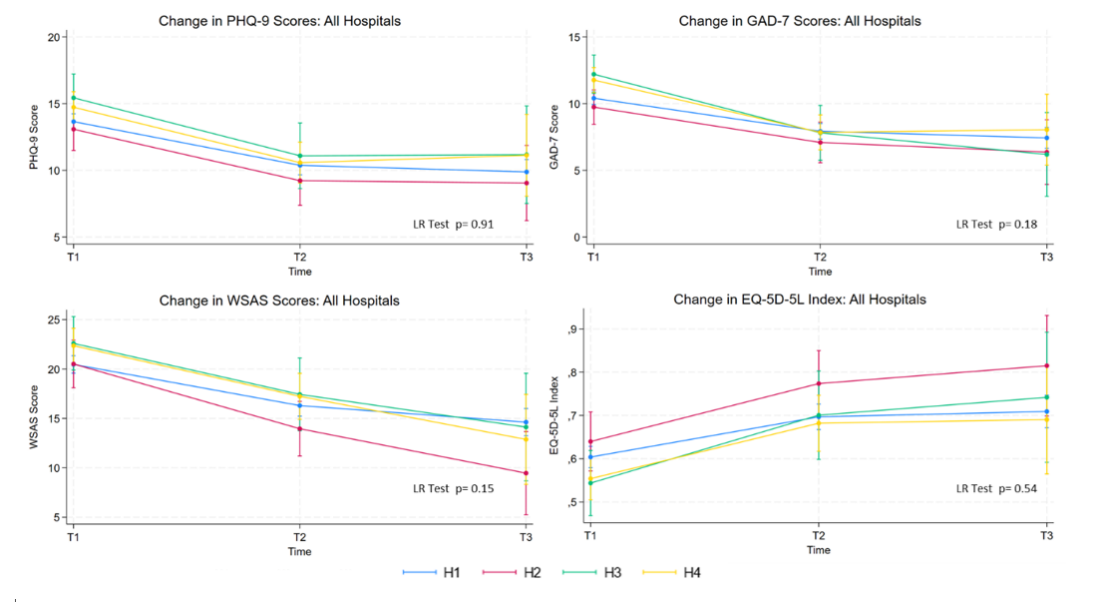
Estimated patient-reported outcomes by hospital over time. GAD-7: 7-item Generalized Anxiety Disorder scale; H1: Haukeland University Hospital; H2: St Olavs Hospital; H3: Vestfold Hospital; H4: Innlandet Hospital; PHQ-9: 9-item Patient Health Questionnaire; T1: baseline; T2: posttreatment; T3: 6-month follow-up; WSAS: Work and Social Adjustment Scale.

Figure 2 illustrates the predicted mean PHQ-9, GAD-7, WSAS, and EQ-5D-5L scores for each hospital at T1, T2, and T3, along with their corresponding 95% CIs and the *P* value from the LR test comparing the hospital-time interaction model with “hospital” as a fixed covariate only. Visually inspecting the plots showed overlapping 95% CIs at T2 or T3 for all the patient-reported outcomes, indicating no difference in the effect between hospitals over time. The LR tests (all *P*>.15) supported this finding. The supplementary analysis, where hospitals were compared based on key characteristics, also did not indicate any difference in the effect between hospitals (Figures S1-S3 in Multimedia Appendix 1).

### Clinically Relevant Effects

We estimated clinically relevant effects using literature-informed cutoffs [45-48,51,52] for response and remission for the PHQ-9, SPIN, PDSS, and WSAS. These are summarized in Table 3. In the depression group, 44/140 (31.4%) patients responded to treatment, while 21/79 (26.6%) patients in the social anxiety group and 37/72 (51.4%) patients in the panic disorder group showed a positive treatment response. Regarding remission, 17/139 (12.2%) patients in the depression group, 26/73 (35.6%) patients in the social anxiety group, and 23/67 (34.3%) patients in the panic disorder group achieved remission, as measured by the PHQ-9, SPIN, and PDSS, respectively. In terms of work and social functioning (WSAS), 97/294 (33.0%) patients positively responded to treatment, whereas 60/261 (23.0%) achieved remission.

**Table 3:** Proportion of patients with clinically relevant symptom reduction.

Response type and group		Outcome	Cutoff; study	Participants, n (%)
**Positive response**				
	Depression (n=140)	PHQ-9^a^	–50%; Centers for Medicare & Medicaid Services [45]	44 (31.4)
	Social anxiety (n=79)	SPIN^b^	–55%; Connor et al [47]	21 (26.6)
	Panic disorder (n=72)	PDSS^c^	–40%; Furukawa et al [48]	37 (51.4)
	All (n=294)	WSAS^d^	–8 points; Rush et al [52]	97 (33.0)
**Negative response**				
	Depression (n=140)	PHQ-9	+50%; Centers for Medicare & Medicaid Services [45]	4 (2.9)
	Social anxiety (n=79)	SPIN	+55%; Connor et al [47]	1 (1.3)
	Panic disorder (n=72)	PDSS	+40%; Furukawa et al [48]	6 (8.3)
	All (n=294)	WSAS	+8 points; Rush et al[52]	15 (5.1)
**No change**				
	Depression (n=140)	PHQ-9	±49%; Centers for Medicare & Medicaid Services [45]	92 (65.7)
	Social anxiety (n=79)	SPIN	±54%; Connor et al [47]	57 (72.2)
	Panic disorder (n=72)	PDSS	±39%; Furukawa et al [48]	29 (40.3)
	All (n=294)	WSAS	±7 points; Rush et al [52]	182 (61.9)
**Remission**				
	Depression (n=139)	PHQ-9	Score≤5; Angstman et al [46]	17 (12.2)
	Social anxiety (n=73)	SPIN	Score≤19; Connor et al [47]	26 (35.6)
	Panic disorder (n=67)	PDSS	Score≤5; Furukawa et al [48]	23 (34.3)
	All (n=261)	WSAS	Score≤10; Mundt et al [51]	60 (23.0)

^a^PHQ-9: 9-item Patient Health Questionnaire.

^b^SPIN: Social Phobia Inventory.

^c^PDSS: Panic Disorder Severity Scale.

^d^WSAS: Work and Social Adjustment Scale.

### Attrition and Adherence

Attrition was assessed in terms of questionnaire completion, while adherence was evaluated in terms of modules completed. Table 4 summarizes the difference in questionnaire completion by demographic characteristics, treatment group, hospital, and module completion. In the overall sample (N=565), 319 (56.5%) participants completed questionnaires at T2, while 129 (22.8%) completed questionnaires at T3.

Among demographic characteristics, questionnaire completion did not depend on gender, civil status, or employment status but was positively associated with age and education. The mean age of the sample at T1 was 32.9 (SD 10.92) years, but those completing T2 and T3 questionnaires were on average 34.2 (SD 11.26) and 35.2 (SD 11.74) years old. In the overall sample, 177 (31.3%) had completed higher education, but among those completing questionnaires at T2 and T3, the share of higher education was 122 (38.2%) and 63 (48.8%), respectively. Self-referred patients were more likely to respond to questionnaires at T2 and T3, while those indicating medication use at T1 were more likely to respond at T3.

The level of questionnaire completion was similar across treatment groups at T2 but varied at T3, with the social anxiety group having the lowest completion rate. Questionnaire completion also differed between hospitals, with H3 and H4 having the lowest completion rates.

Patients completed a mean of 4.6 (SD 2.39) modules (depression: mean 4.6, SD 2.33; social anxiety disorder: mean 4.4, SD 2.51; panic disorder: mean 4.7, SD 2.37), which varied between hospitals (H1: mean 4.3, SD 2.32; H2: mean 5.08, SD 2.53; H3: mean 5.7, SD 2.43; H4: mean 4.6, SD 2.42). Treatment completion was achieved by 54.2% (294/542) of patients (depression: 165/260, 63.5%; social anxiety disorder: 64/150, 42.7%, panic disorder: 65/132, 49.2%), whereas 12.9% (70/542) completed all the treatment modules (depression: 43/260, 16.5%; social anxiety disorder: 15/150, 10%; panic disorder: 12/132, 9.1%). Module and questionnaire completion were positively associated. Participants who completed the T2 assessment completed on average 5.7 (SD 2.10) modules, while those who completed the T3 assessment completed on average 6.1 (SD 2.11) modules. In contrast, participants who did not complete the T2 or the T3 assessment completed only 3.1 (SD 1.94) and 4.1 (SD 2.28) modules on average, respectively. Supplementary analysis showed that treatment completion in terms of modules completed yielded similar results to the main analyses. Exploring different scenarios for data being missing not at random indicated that patients experienced improvement in all outcomes at all time points, except for EQ-5D-5L at 30% delta adjustment, where there was no change in outcomes at T3 compared to T1 (Tables S4 and S5 in Multimedia Appendix 1).

**Table 4:** Questionnaire completion at T2^a^ and T3^b^ time points.

Characteristics		T1^c^ (N=565), n (%)	T2 (n=319), n (%)	T3 (n=129), n (%)
**Demographics**				
	Female	356 (63.0)	205 (64.3)	75 (58.1)
	Married/cohabiting	198 (35.0)	124 (38.9)	52 (40.3)
	Completed higher education	177 (31.3)	122 (38.2)	63 (48.8)
	Employed or student	425 (75.2)	245 (76.8)	103 (79.8)
	Self-referred	151 (26.7)	102 (32.0)	81 (62.8)
	Using medicine	237 (41.9)	124 (38.9)	41 (31.8)
**Treatment group**				
	Depression	271 (48.0)	154 (48.3)	58 (45.0)
	Social anxiety	157 (27.8)	86 (27.0)	29 (22.5)
	Panic disorder	137 (24.2)	79 (24.8)	42 (32.6)
**Hospital**				
	H1^d^	376 (66.5)	221 (69.3)	104 (80.6)
	H2^e^	52 (9.2)	34 (10.7)	10 (7.8)
	H3^f^	41 (7.3)	18 (5.6)	7 (5.4)
	H4^g^	96 (17.0)	46 (14.4)	8 (6.2)
**Modules completed**				
	All	70 (12.4)	62 (19.4)	31 (24.0)
	≥4	361 (63.9)	263 (82.4)	113 (87.6)

^a^T2: posttreatment

^b^T3: 6-month follow-up

^c^T1: baseline

^d^H1: Haukeland University Hospital.

^e^H2: St Olavs Hospital.

^f^H3: Vestfold Hospital.

^g^H4: Innlandet Hospital.

### Costs

The total mean program costs per patient, as presented in Table 5, showed major differences between hospitals. Costs varied from US $636.41 per patient at H4 to US $2152.47 per patient at H2. To highlight the impact of the fixed annual infrastructure costs versus the patient-dependent therapist costs, separate costs were presented for patients starting treatment in 2022 or earlier versus patients who started treatment in 2023 or later. The 2022 and 2023 columns show that the total mean program costs largely comprised infrastructure costs per patient, which are influenced by the volume of patients treated each year. This can be illustrated for H2, where the number of patients decreased by 51% from 2022 to 2023, causing the infrastructure costs to increase from US $1414.66 to US $2893.19 per patient (Table 5). For H1 and H3, minor changes were observed between 2022 and 2023. In H4, only 2023 costs were presented, since the inclusion of study participants started in 2023. The detailed subdivision of infrastructure costs into maintenance, implementation, and number of patients treated can be found in Table S6 in Multimedia Appendix 1.

**Table 5:** Program costs per patient (2022 NOK^a^-US $ exchange rate) by year and by hospital.

Years and hospitals		Participants, n (%)	Infrastructure cost (US $), mean (SD)	Therapist guidance cost (US $), mean (SD)	Total cost (US $), mean (SD)	
**All years**						
	H1^b^	376 (66.5)	782.66 (75.3)	229.47 (67.2)	1012.12 (104.0)	
	H2^c^	52 (9.2)	1898.02 (700.3)	254.43 (65.7)	2152.47 (701.4)	
	H3^d^	41 (7.3)	435.16 (13.6)	258.29 (76.9)	693.45 (78.0)	
	H4^e^	96 (17.0)	401.68 (0.0)	234.73 (71.5)	636.41 (71.5)	
	Total	565 (100)	795.37 (442.9)	234.75 (69.0)	1030.12 (451.6)	
**2022**						
	H1	169 (79.0)	865.89 (0.0)	234.13 (63.4)	1100.02 (63.4)	
	H2	35 (16.4)	1414.66 (0.0)	255.84 (66.6)	1670.51 (66.6)	
	H3	10 (4.7)	458.8 (0.0)	257.45 (81.9)	716.25 (81.9)	
	H4	N/A^f^	N/A	N/A	N/A	
	Total	214 (100)	936.63 (228.6)	238.77 (65.2)	1175.39 (242.6)	
**2023**						
	H1	207 (59.0)	714.7 (0.0)	225.66 (70.0)	940.37 (70.0)	
	H2	17 (4.8)	2893.19 (0.0)	251.56 (65.6)	3144.74 (65.6)	
	H3	31 (8.8)	427.54 (0.0)	258.56 (76.6)	686.10 (76.6)	
	H4	96 (27.4)	401.68 (0.0)	234.73 (71.5)	636.41 (71.5)	
	Total	351 (100)	709.24 (514.5)	232.30 (71.2)	941.54 (521.6)	

^a^NOK: Norwegian kroner; an exchange rate of US $1=NOK 9.62 in 2022 was applied.

^b^H1: Haukeland University Hospital.

^c^H2: St Olavs Hospital.

^d^H3: Vestfold Hospital.

^e^H4: Innlandet Hospital.

^f^N/A: not applicable; inclusion of patients in H4 started in 2023.

Therapist costs per patient showed a slight variation, both between hospitals and within hospitals (ie, 2022 vs 2023). Overall, the mean therapist costs per patient varied from US $ 229.47 at H1 to US $258.29 at H3 per patient between hospitals, whereas small differences were observed between years (2022: US $234.13-$257.45 per patient; 2023: US $225.66-$258.56 per patient), as shown in Table 5. The therapist cost differences can be attributed to both the hospital-specific therapist unit price per hour and the number of modules completed per patient. The therapist unit price per hour is detailed by hospital and by year in Table S6 in Multimedia Appendix 1.

## Discussion

### Principal Findings

This study evaluated the costs and effects of therapist-guided iCBT (eCoping) in routine specialist care, comparing outcomes between health regions in Norway. Our sample, representative of patients in specialist outpatient mental health services in Norway [61], achieved reductions in symptom severity, improvements in functioning, and enhancements in the HRQoL, with effects sustained at the 6-month follow-up. These results were largely consistent between hospitals. Therapist costs per patient were also comparable, but we found notable differences in infrastructure costs per patient, mainly due to the number of patients treated in each hospital. To the best of our knowledge, this is the first study to examine the costs and outcomes of an iCBT program between hospitals over such a broad range of outcomes.

Comparing the symptom severity outcomes from our study to previous studies, we found mixed results. Social anxiety treatment performed comparably to a previous single-site evaluation of eCoping [33], whereas depression and panic disorder treatments had slightly lower effects [32,39]. Similarly, comparing our study’s outcomes with those of other iCBT treatments in routine care, we found that patients with panic disorder and social anxiety experienced improvements consistent with published reports [62,63], but eCoping’s effect on depression was somewhat lower [62]. Overall, our sample had lower treatment adherence compared to previous studies [32,62]. That could possibly explain the observed difference, although treatment completion did not alter clinical outcomes for our sample (Table S3 in Multimedia Appendix 1). Unsurprisingly, our study’s effectiveness is also lower when compared to similar iCBT treatments evaluated in RCTs [5,64], but that is expected due to stricter protocols and the “adherence fostering” effect typically seen in controlled trial settings [62]. Despite these differences, eCoping is more effective compared to the reported effectiveness of treatment-as-usual (TAU) in Nordic countries [65] and demonstrates similar outcomes compared to other evidence-based treatments in routine care [66].

Beyond improvements in symptom severity, eCoping was associated with substantial gains in work adjustment, social functioning, and the HRQoL. The change in WSAS scores aligns with the effectiveness of other evidence-based interventions [67]. The HRQoL also improved in line with clinical outcomes, with EQ-5D-5L index gains observed across all disorders. These outcomes are comparable to similar interventions in mental health care [68].

Norway uses a decentralized model for guided iCBT implementation, allowing hospitals to independently opt in to the eCoping program and to tailor its organization to local needs. Despite variations in referral pathways (eg, self-referral vs GP-only referral), service history, patient volume, therapist skill mix, and capacity [69], eCoping demonstrated consistent effectiveness across the 4 participating hospitals in the main and supplementary analyses. This indicates that therapist-guided iCBT, when scaled from a single-unit offer to a national program, can deliver consistent outcomes in new locations.

From a cost standpoint, we observed consistent therapist costs per patient between hospitals but large variations in infrastructure costs per patient. In line with the previous literature [29,30], patient volume was the primary factor influencing fixed infrastructure costs per patient. In our study, H2 struggled with patient recruitment, possibly due to implementation challenges, and experienced a large increase in infrastructure costs per patient from 2022 to 2023. This highlights the challenges of integrating iCBT into traditional hospital settings and has implications for both cost-effectiveness and implementation. Gega et al [70] highlighted that patient recruitment costs are rarely considered in economic evaluations, despite being a key determinant of an intervention’s cost-effectiveness. Variations in recruitment rates across hospitals further emphasize the need for hybrid-design studies to assess strategies that enhance the program’s uptake and efficient use of resources. Although direct cost comparisons with previous studies are difficult due to methodological differences, our therapist costs (US $234.75 per patient) were lower than prior estimates (US $140.31-$2929.94; n=8), while total costs, including infrastructure (US $1030.12 per patient), were somewhat higher (US $218.12-$1530.41; n=4) [71].

From a societal perspective, it is of interest to compare program costs to the observed reduction in sickness absence. Participants reported an average reduction of 3.2 days in sickness-related absence posttreatment and 7.7 days at 6-month follow-up—a large improvement compared to similar interventions [72]. Applying the approach of Aasdahl et al [73], the observed sick leave change corresponded to an average productivity gain of US $1166.35 per month posttreatment and US $2825.07 per month at 6-month follow-up. Essentially, the program cost of US $1030.12 per patient seems to be recovered by a wide margin with the observed change in productivity.

### Limitations

This study has several limitations. The lack of a control group precludes direct evaluation of eCoping’s effectiveness and cost-effectiveness relative to a comparator and precludes causal interpretations of effects. Additionally, low data completion and program adherence may have reduced the precision of our results and introduced bias. Although we maximized the use of the available data for hospital-level analyses, large variations in sample sizes across hospitals, paired with a lack of information about therapist expertise and implementation fidelity, may have limited our ability to detect differences between hospitals. We are cognizant that our findings are context specific and may not be generalizable to other health care settings. Future studies could benefit from participation from more hospitals and a control arm for comparison.

### Conclusion

This study demonstrates that eCoping in routine specialist care shows satisfactory improvements in symptom reduction, functioning, and the HRQoL, with sustained effects at 6-month follow-up. Notably, these results were consistent across hospitals. The therapists’ costs between hospitals were also comparable, but the infrastructure costs per patient varied due to differences in patient volume. Addressing challenges related to adherence and patient recruitment could further enhance the program’s effectiveness and efficient use of resources. We hope that our study highlights the importance of reviewing local variation in both costs and outcomes in order to achieve successful implementation of therapist-guided iCBT.

## Appendix

Multimedia Appendix 1 Program costs, patient-reported outcomes over time, patient-reported outcomes by hospital, and Stata code.

## Abbreviations

GAD-7: 7-item Generalized Anxiety Disorder scale
GP: general practitioner
H1: Haukeland University Hospital
H2: St Olavs Hospital
H3: Vestfold Hospital
H4: Innlandet Hospital
HRQoL: health-related quality of life
iCBT: internet-delivered cognitive behavioral therapy
LR: likelihood ratio
NOK: Norwegian kroner
PDSS: Panic Disorder Severity Scale
PHQ-9: 9-item Patient Health Questionnaire
RCT: randomized clinical trial
SPIN: Social Phobia Inventory
T1: baseline
T2: posttreatment
T3: 6-month follow-up
WSAS: Work and Social Adjustment Scale

## References

[1] Lattie EG, Stiles-Shields C, Graham AK (2022). An overview of and recommendations for more accessible digital mental health services. Nat Rev Psychol.

[2] Titov N, Hadjistavropoulos HD, Nielssen O, Mohr DC, Andersson G, Dear BF (2019). From research to practice: ten lessons in delivering digital mental health services. JCM.

[3] Faria M, Zin STP, Chestnov R, Novak AM, Lev-Ari S, Snyder M (2023). Mental health for all: the case for investing in digital mental health to improve global outcomes, access, and innovation in low-resource settings. JCM.

[4] Chow DY, Jiang X, You JH (2022). Information technology-based versus face-to-face cognitive-behavioural therapy for anxiety and depression: a systematic review and meta-analysis. J Affect Disorders.

[5] Karyotaki E (2021). Internet-based cognitive behavioral therapy for depression: a systematic review and individual patient data network meta-analysis. JAMA Psychiatry.

[6] Esfandiari N, Mazaheri M, Akbari-Zardkhaneh S, Sadeghi-Firoozabadi V, Cheraghi M (2021). Internet-delivered versus face-to-face cognitive behavior therapy for anxiety disorders: systematic review and meta-analysis. Int J Prev Med.

[7] Pauley D, Cuijpers P, Papola D, Miguel C, Karyotaki E (2021). Two decades of digital interventions for anxiety disorders: a systematic review and meta-analysis of treatment effectiveness. Psychol Med.

[8] Papola D, Ostuzzi G, Tedeschi F, Gastaldon C, Purgato M, Del Giovane C, Pompoli A, Pauley D, Karyotaki E, Sijbrandij M, Furukawa TA, Cuijpers P, Barbui C (2022). CBT treatment delivery formats for panic disorder: a systematic review and network meta-analysis of randomised controlled trials. Psychol Med.

[9] Luo C, Sanger N, Singhal N, Pattrick K, Shams I, Shahid H, Hoang P, Schmidt J, Lee J, Haber S, Puckering M, Buchanan N, Lee P, Ng K, Sun S, Kheyson S, Chung DC, Sanger S, Thabane L, Samaan Z (2020). A comparison of electronically-delivered and face to face cognitive behavioural therapies in depressive disorders: a systematic review and meta-analysis. eClinicalMedicine.

[10] Kolovos S, van Dongen JM, Riper H, Buntrock C, Cuijpers P, Ebert DD, Geraedts AS, Kenter RM, Nobis S, Smith A, Warmerdam L, Hayden JA, van Tulder MW, Bosmans JE (2018). Cost effectiveness of guided Internet-based interventions for depression in comparison with control conditions: an individual-participant data meta-analysis. Depress Anxiety.

[11] Kählke F, Buntrock C, Smit F, Ebert DD (2022). Systematic review of economic evaluations for internet- and mobile-based interventions for mental health problems. npj Digit Med.

[12] Rohrbach PJ, Dingemans AE, Evers C, Van Furth EF, Spinhoven P, Aardoom JJ, Lähde I, Clemens FC, Van den Akker-Van Marle ME (2023). Cost-effectiveness of internet interventions compared with treatment as usual for people with mental disorders: systematic review and meta-analysis of randomized controlled trials. J Med Internet Res.

[13] Jenkins-Guarnieri MA, Pruitt LD, Luxton DD, Johnson K (2015). Patient perceptions of telemental health: systematic review of direct comparisons to in-person psychotherapeutic treatments. Telemed e-Health.

[14] Molloy A, Ellis DM, Su L, Anderson PL (2021). Improving acceptability and uptake behavior for internet-based cognitive-behavioral therapy. Front Digit Health.

[15] NHS Confederation’s Mental Health Network (2023). Maximising the potential of digital in mental health. NHS Confederation.

[16] Titov N, Dear B, Nielssen O, Staples L, Hadjistavropoulos H, Nugent M, Adlam K, Nordgreen T, Bruvik KH, Hovland A, Repål A, Mathiasen K, Kraepelien M, Blom K, Svanborg C, Lindefors N, Kaldo V (2018). ICBT in routine care: a descriptive analysis of successful clinics in five countries. Internet Intervent.

[17] Worm-Smeitink M, van Dam A, van Es S, van der Vaart R, Evers A, Wensing M, Knoop H (2019). Internet-based cognitive behavioral therapy for chronic fatigue syndrome integrated in routine clinical care: implementation study. J Med Internet Res.

[18] Bucci S, Schwannauer M, Berry N (2019). The digital revolution and its impact on mental health care. Psychol Psychother.

[19] Van Spall HGC, Toren A, Kiss A, Fowler RA (2007). Eligibility criteria of randomized controlled trials published in high-impact general medical journals: a systematic sampling review. JAMA.

[20] Drozd F, Vaskinn L, Bergsund HB, Haga SM, Slinning K, Bjørkli CA (2016). The implementation of internet interventions for depression: a scoping review. J Med Internet Res.

[21] Vis C, Kleiboer A, Mol M, Pedersen CD, Finch T, Smit J, Riper H, Albaina O, Cavallo M, Dozeman E, Duedal Pedersen C, Ebert D, Etzelmüller A, van der Eycken E, Fullaondo A, Gabilondo A, González Pinto A, Gutiérrez B, Kleiboer A, Kohls E, de Manuel E, Mathiasen K, Mol M, Mora J, Peleteiro-Pensado L, Ponte J, Power K, Retolaza A, Riper H, Sacco Y, van Schaik A, Sierra Callau M, Skjøth MM, Smit J, Sogomonjan M, Tajes-Alonso M, Txarramendieta J, Vis C, Wright C, Zanalda E (2022). Organisational implementation climate in implementing internet-based cognitive behaviour therapy for depression. BMC Health Serv Res.

[22] Maqsood A, Gul S, Zahra T, Noureen N, Khattak A (2024). From face-to-face to screen-to-screen: exploring the multifaceted dimensions of digital mental health care. Front Psychiatry.

[23] Bucci S, Berry N, Morris R, Berry K, Haddock G, Lewis S, Edge D (2019). “They are not hard-to-reach clients. We have just got hard-to-reach services.” Staff views of digital health tools in specialist mental health services. Front Psychiatry.

[24] Patel S, Akhtar A, Malins S, Wright N, Rowley E, Young E, Sampson S, Morriss R (2020). The acceptability and usability of digital health interventions for adults with depression, anxiety, and somatoform disorders: qualitative systematic review and meta-synthesis. J Med Internet Res.

[25] Berardi C, Antonini M, Jordan Z, Wechtler H, Paolucci F, Hinwood M (2024). Barriers and facilitators to the implementation of digital technologies in mental health systems: a qualitative systematic review to inform a policy framework. BMC Health Serv Res.

[26] Gullickson KM, Hadjistavropoulos HD, Dear BF, Titov N (2019). Negative effects associated with internet-delivered cognitive behaviour therapy: an analysis of client emails. Internet Intervent.

[27] Drummond MF, Sculpher MJ, Claxton K, Stoddart GL, Torrance GW (2015). Methods for the Economic Evaluation of Health Care Programmes. Fourth Edition.

[28] Jankovic D, Bojke L, Marshall D, Saramago Goncalves P, Churchill R, Melton H, Brabyn S, Gega L (2020). Systematic review and critique of methods for economic evaluation of digital mental health interventions. Appl Health Econ Health Policy.

[29] Gomes M, Murray E, Raftery J (2022). Economic evaluation of digital health interventions: methodological issues and recommendations for practice. PharmacoEconomics.

[30] McNamee P, Murray E, Kelly MP, Bojke L, Chilcott J, Fischer A, West R, Yardley L (2016). Designing and undertaking a health economics study of digital health interventions. Am J Prev Med.

[31] Kidholm K, Clemensen J, Caffery LJ, Smith AC (2017). The Model for Assessment of Telemedicine (MAST): a scoping review of empirical studies. J Telemed Telecare.

[32] Nordgreen T, Blom K, Andersson G, Carlbring P, Havik OE (2019). Effectiveness of guided internet-delivered treatment for major depression in routine mental healthcare - an open study. Internet Intervent.

[33] Nordgreen T, Gjestad R, Andersson G, Carlbring P, Havik OE (2018). The effectiveness of guided internet-based cognitive behavioral therapy for social anxiety disorder in a routine care setting. Internet Intervent.

[34] Nye Metoder (2019). Beslutningsforum for nye metoder. The National System for Managed Introduction of New Health Technologies within the Specialist Health Service in Norway.

[35] Andersson G, Bergström J, Holländare F, Carlbring P, Kaldo V, Ekselius L (2018). Internet-based self-help for depression: randomised controlled trial. Br J Psychiatry.

[36] Furmark T, Carlbring P, Hedman E, Sonnenstein A, Clevberger P, Bohman B, Eriksson A, Hållén A, Frykman M, Holmström A, Sparthan E, Tillfors M, Ihrfelt EN, Spak M, Eriksson A, Ekselius L, Andersson G (2018). Guided and unguided self-help for social anxiety disorder: randomised controlled trial. Br J Psychiatry.

[37] Paxling B, Almlöv J, Dahlin M, Carlbring P, Breitholtz E, Eriksson T, Andersson G (2011). Guided internet-delivered cognitive behavior therapy for generalized anxiety disorder: a randomized controlled trial. Cogn Behav Ther.

[38] Nordgren LB, Hedman E, Etienne J, Bodin J, Eriksson S, Lindkvist E, Andersson G, Carlbring P, Kadowaki (2014). Effectiveness and cost-effectiveness of individually tailored Internet-delivered cognitive behavior therapy for anxiety disorders in a primary care population: a randomized controlled trial. Behav Res Ther.

[39] Nordgreen T, Haug T, Öst L, Andersson G, Carlbring P, Kvale G, Tangen T, Heiervang E, Havik OE (2016). Stepped care versus direct face-to-face cognitive behavior therapy for social anxiety disorder and panic disorder: a randomized effectiveness trial. Behav Ther.

[40] Ørjasæter Elvsaas I-K, Stoinska‐Schneider A, Smedslund G (2018). Therapist‐supported internet therapy for mental disorders – a health technology assessment. Folkehelseinstituttet.

[41] CheckWare AS.

[42] Kroenke K, Spitzer RL, Williams JBW (2001). The PHQ-9: validity of a brief depression severity measure. J Gen Intern Med.

[43] Beard C, Hsu K, Rifkin L, Busch A, Björgvinsson T (2016). Validation of the PHQ-9 in a psychiatric sample. J Affect Disorders.

[44] Titov N, Dear BF, McMillan D, Anderson T, Zou J, Sunderland M (2011). Psychometric comparison of the PHQ-9 and BDI-II for measuring response during treatment of depression. Cogn Behav Ther.

[45] (2017). Depression remission at twelve months. Centers for Medicare & Medicaid Services.

[46] Angstman KB, Pietruszewski P, Rasmussen NH, Wilkinson JM, Katzelnick DJ (2012). Depression remission after six months of collaborative care management: role of initial severity of depression in outcome. Ment Health Fam Med.

[47] Connor KM, Davidson JRT, Churchill LE, Sherwood A, Weisler RH, Foa E (2018). Psychometric properties of the Social Phobia Inventory (SPIN): new self-rating scale. Br J Psychiatry.

[48] Furukawa TA, Katherine Shear M, Barlow DH, Gorman JM, Woods SW, Money R, Etschel E, Engel RR, Leucht S (2008). Evidence‐based guidelines for interpretation of the Panic Disorder Severity Scale. Depress Anxiety.

[49] Spitzer RL, Kroenke K, Williams JBW, Löwe B (2006). A brief measure for assessing generalized anxiety disorder: the GAD-7. Arch Intern Med.

[50] Toussaint A, Hüsing P, Gumz A, Wingenfeld K, Härter M, Schramm E, Löwe B (2020). Sensitivity to change and minimal clinically important difference of the 7-item Generalized Anxiety Disorder Questionnaire (GAD-7). J Affect Disorders.

[51] Mundt JC, Marks IM, Shear MK, Greist JM (2018). The Work and Social Adjustment Scale: a simple measure of impairment in functioning. Br J Psychiatry.

[52] Rush AJ, South C, Jain S, Agha R, Zhang M, Shrestha S, Khan Z, Hassan M, Trivedi MH (2021). Clinically significant changes in the 17- and 6-item Hamilton Rating Scales for Depression: a STAR*D report. Neuropsychiatr Dis Treat.

[53] EQ-5D-5L. EuroQol Group.

[54] Garratt AM, Stavem K, Shaw JW, Rand K (2024). EQ-5D-5L value set for Norway: a hybrid model using cTTO and DCE data. Qual Life Res.

[55] Khan ZA, Kidholm K, Pedersen SA, Haga SM, Drozd F, Sundrehagen T, Olavesen E, Halsteinli V (2024). Developing a program costs checklist of digital health interventions: a scoping review and empirical case study. PharmacoEconomics.

[56] Inflation calculator. Statistics Norway.

[57] Exchange rates. Norges Bank (Central Bank of Norway).

[58] StataNow. STATA.

[59] Faria R, Gomes M, Epstein D, White IR (2014). A guide to handling missing data in cost-effectiveness analysis conducted within randomised controlled trials. PharmacoEconomics.

[60] Huque MH, Carlin JB, Simpson JA, Lee KJ (2018). A comparison of multiple imputation methods for missing data in longitudinal studies. BMC Med Res Methodol.

[61] Havnen A, Lindberg MS, Lundqvist J, Brattmyr M, Hjemdal O, Solem S (2024). Health-related quality of life in psychiatric outpatients: a cross-sectional study of associations with symptoms, diagnoses, and employment status. Qual Life Res.

[62] Etzelmueller A, Vis C, Karyotaki E, Baumeister H, Titov N, Berking M, Cuijpers P, Riper H, Ebert DD (2020). Effects of internet-based cognitive behavioral therapy in routine care for adults in treatment for depression and anxiety: systematic review and meta-analysis. J Med Internet Res.

[63] Efron G, Wootton BM (2021). Remote cognitive behavioral therapy for panic disorder: a meta-analysis. J Anxiety Disorders.

[64] Kambeitz-Ilankovic L, Rzayeva U, Völkel L, Wenzel J, Weiske J, Jessen F, Reininghaus U, Uhlhaas PJ, Alvarez-Jimenez M, Kambeitz J (2022). A systematic review of digital and face-to-face cognitive behavioral therapy for depression. npj Digit Med.

[65] Brattmyr M, Lindberg MS, Lundqvist J, Öst L, Solem S, Hjemdal O, Havnen A (2023). Clinically representative therapy for Nordic adult outpatients with common mental health problems: a systematic review and meta‐analysis. Scand J Psychol.

[66] Knapstad M, Nordgreen T, Smith ORF (2018). Prompt mental health care, the Norwegian version of IAPT: clinical outcomes and predictors of change in a multicenter cohort study. BMC Psychiatry.

[67] Wakefield S, Kellett S, Simmonds‐Buckley M, Stockton D, Bradbury A, Delgadillo J (2020). Improving Access to Psychological Therapies (IAPT) in the United Kingdom: a systematic review and meta‐analysis of 10‐years of practice‐based evidence. Br J Clin Psychol.

[68] Bratberg G, Leira K, Granan L, Jonsbu E, Fadnes BL, Thuland SF, Myklebust (2020). Learning oriented physiotherapy (LOP) in anxiety and depression: an 18 months multicentre randomised controlled trial (RCT). Eur J Physiother.

[69] Heggelund J, Kidholm K, Halsteinli V (2024). Implementation of guided internet-delivered treatment in Norway: a comparative study of service delivery in four hospitals.

[70] Gega L, Jankovic D, Saramago P, Marshall D, Dawson S, Brabyn S, Nikolaidis GF, Melton H, Churchill R, Bojke L (2022). Digital interventions in mental health: evidence syntheses and economic modelling. Health Technol Assess.

[71] Mitchell LM, Joshi U, Patel V, Lu C, Naslund JA (2021). Economic evaluations of internet-based psychological interventions for anxiety disorders and depression: a systematic review. J Affect Disorders.

[72] Xu H, Cai J, Sawhney R, Jiang S, Buys N, Sun J (2023). The effectiveness of cognitive-behavioral therapy in helping people on sick leave to return to work: a systematic review and meta-analysis. J Occup Rehabil.

[73] Aasdahl L, Fimland MS, Bjørnelv GM, Johnsen R, Vasseljen O, Halsteinli V, Gismervik (2023). Economic evaluation of inpatient multimodal occupational rehabilitation vs. outpatient acceptance and commitment therapy for sick-listed workers with musculoskeletal- or common mental disorders. J Occup Rehabil.

