# Supplementary material for: Costs and Outcomes of a Therapist-Guided Internet-Delivered Cognitive Behavioral Therapy: Multicenter Observational Study
Source: J Med Internet Res. 2025 Jul 28;27:e73067. doi: 10.2196/73067 (PMC12340462; doi:10.2196/73067)
Supplement: Multimedia Appendix 1 [file jmir_v27i1e73067_app1.docx]

### **Patient-Reported Outcomes Over Time**

We performed several supplementary analyses to further investigate change in patient-reported outcomes over time. In Table S1 we summarize all patient-reported outcomes for all treatment groups. In Table S2 we supplement our main analyses by including additional covariates in the mixed models (i.e. patients’ referral pathway, employment status at baseline, and medication use at baseline). These covariares are in addition to patients’ age, gender and hospital, which were included as fixed effects in the main analyses. In Table S3, we present outcomes over time for patients who completed the treatment. Treatment completion is defined as completing 4 out of 8 modules in depression group and 5 out of 9 modules in the social anxiety and panic disorder groups.

We also investigated the effect of different missing data scenarios by first imputing our missing observations using chained equations (MICE). Our imputation model included all outcomes related to symptom severity (PHQ-9, GAD-7, SPIN and PDSS), work and social functioning (WSAS), and health related quality of life (EQ-5D-5L), in addition to patients’ age, gender, employment status, medication use, referral pathway, module completion and hospital of treatment for all time points. Missing observations were matched using predictive mean matching with fifth nearest neighbor. We then adjusted the imputed values by 10%, 20% and 30% to account for scenarios that patients with missing data experienced worse outcomes than the ones who completed the questionnaires. Delta adjustment was only applied to T2 and T3 time points, while T1 values were left unadjusted. The results from the MICE model and delta-adjusted scenarios are presented in Tables S4 and S5.

These sensitivity analyses indicate that patients experienced in all outcomes across the treatment groups (Table S1). Controlling for additional covariates did not alter the outcomes compared to the main analysis (Table S2). Patients who completed treatment experienced similar results to those in the intention-to-treat analysis (Table S3). Mixed model estimates from the imputed dataset were similar to the observed (unimputed) dataset (Table S4). When considering a scenario that data is not missing at random, increasing the assumption of worseness from 10% to 30% reduced treatment effectiveness compared to the main analysis. However, with the exception of EQ-5D-5L at the 30% delta adjustment, the treatment remained effective for all outcomes at all time points with delta adjustments ranging from 10% to 30% (Table S5).

**Table S1.** Observed and predicted patient-reported outcomes over time for all treatment groups.

|  |  |  |  |  |  |  |  |  |  |
| --- | --- | --- | --- | --- | --- | --- | --- | --- | --- |
|  | | | **Descriptive** | | | **Regression Coefficient for Time** | | | |
| **Group^a^** | **Outcome** | **Time point** | **n** | **Mean** | **SD** | **Estimate^b^** | **95% CI** | **p** | **ES^c^** |
| Dep | PHQ-9 | T1 | 267 | 15.9 | 5.11 |  |  |  |  |
| Dep | PHQ-9 | T2 | 143 | 11.1 | 6.04 | -4.4 | -5.2 to -3.6 | <0.001 | -0.85 |
| Dep | PHQ-9 | T3 | 56 | 9.1 | 5.42 | -5.5 | -6.7 to -4.3 | <0.001 | -1.08 |
| Dep | GAD-7 | T1 | 266 | 10.1 | 4.68 |  |  |  |  |
| Dep | GAD-7 | T2 | 141 | 7.9 | 4.76 | -1.9 | -2.6 to -1.3 | <0.001 | -0.41 |
| Dep | GAD-7 | T3 | 58 | 6.5 | 3.78 | -2.4 | -3.4 to -1.5 | <0.001 | -0.52 |
| Dep | WSAS | T1 | 268 | 22.7 | 7.40 |  |  |  |  |
| Dep | WSAS | T2 | 146 | 17.7 | 9.15 | -4.6 | -5.7 to -3.4 | <0.001 |  |
| Dep | WSAS | T3 | 56 | 13.3 | 9.14 | -7.1 | -8.8 to -5.3 | <0.001 |  |
| Dep | EQ-5D-5L | T1 | 268 | 0.6 | 0.24 |  |  |  |  |
| Dep | EQ-5D-5L | T2 | 150 | 0.7 | 0.21 | 0.08 | 0.05 to 0.11 | <0.001 |  |
| Dep | EQ-5D-5L | T3 | 58 | 0.8 | 0.19 | 0.09 | 0.05 to 0.13 | <0.001 |  |
| SAD | PHQ-9 | T1 | 155 | 12.6 | 5.68 |  |  |  |  |
| SAD | PHQ-9 | T2 | 81 | 9.1 | 5.94 | -2.5 | -3.6 to -1.4 | <0.001 | -0.44 |
| SAD | PHQ-9 | T3 | 28 | 8.2 | 5.68 | -2.3 | -3.9 to -0.6 | 0.007 | -0.40 |
| SAD | GAD-7 | T1 | 155 | 10.8 | 4.73 |  |  |  |  |
| SAD | GAD-7 | T2 | 81 | 7.1 | 4.84 | -3.3 | -4.3 to -2.2 | <0.001 | -0.69 |
| SAD | GAD-7 | T3 | 28 | 6.3 | 4.54 | -3.7 | -5.3 to -2.1 | <0.001 | -0.78 |
| SAD | WSAS | T1 | 155 | 20.3 | 8.21 |  |  |  |  |
| SAD | WSAS | T2 | 80 | 14.6 | 10.11 | -5.0 | -6.7 to -3.4 | <0.001 | -0.61 |
| SAD | WSAS | T3 | 28 | 11.9 | 8.69 | -6.5 | -9.0 to -3.9 | <0.001 | -0.79 |
| SAD | EQ-5D-5L | T1 | 268 | 0.6 | 0.25 |  |  |  |  |
| SAD | EQ-5D-5L | T2 | 150 | 0.7 | 0.24 | 0.11 | 0.07 to 0.15 | <0.001 |  |
| SAD | EQ-5D-5L | T3 | 58 | 0.8 | 0.24 | 0.09 | 0.03 to 0.15 | 0.003 |  |
| SAD | SPIN | T1 | 155 | 40.3 | 11.87 |  |  |  |  |
| SAD | SPIN | T2 | 81 | 26.6 | 15.41 | -13.2 | -16.0 to -10.4 | <0.001 | -1.11 |
| SAD | SPIN | T3 | 28 | 22.4 | 15.86 | -15.5 | -19.9 to -11.1 | <0.001 | -1.30 |
| PD | PHQ-9 | T1 | 136 | 11.7 | 5.74 |  |  |  |  |
| PD | PHQ-9 | T2 | 74 | 7.8 | 5.77 | -3.1 | -4.0 to -2.2 | <0.001 | -0.54 |
| PD | PHQ-9 | T3 | 42 | 7.8 | 6.25 | -2.6 | -3.8 to -1.4 | <0.001 | -0.46 |
| PD | GAD-7 | T1 | 136 | 11.8 | 4.99 |  |  |  |  |
| PD | GAD-7 | T2 | 75 | 7.4 | 4.40 | -4.0 | -5 to -3.1 | <0.001 | -0.80 |
| PD | GAD-7 | T3 | 42 | 6.8 | 4.43 | -4.3 | -5.5 to -3.1 | <0.001 | -0.86 |
| PD | WSAS | T1 | 136 | 18.3 | 9.05 |  |  |  |  |
| PD | WSAS | T2 | 73 | 13.2 | 10.22 | -4.4 | -6 to -2.9 | <0.001 | -0.49 |
| PD | WSAS | T3 | 42 | 10.9 | 8.93 | -6.2 | -8.1 to -4.3 | <0.001 | -0.69 |
| PD | EQ-5D-5L | T1 | 132 | 0.6 | 0.28 |  |  |  |  |
| PD | EQ-5D-5L | T2 | 76 | 0.7 | 0.26 | 0.11 | 0.06 to 0.16 | 0.001 |  |
| PD | EQ-5D-5L | T3 | 42 | 0.8 | 0.19 | 0.15 | 0.08 to 0.21 | <0.001 |  |
| PD | PDSS | T1 | 136 | 12.3 | 5.52 |  |  |  |  |
| PD | PDSS | T2 | 73 | 7.8 | 5.35 | -4.4 | -5.5 to -3.3 | <0.001 | -0.79 |
| PD | PDSS | T3 | 42 | 6.5 | 4.99 | -5.3 | -6.7 to -4 | <0.001 | -0.96 |
| All | Sick leave (days) | T1 | 267 | 15.9 | 5.11 |  |  |  |  |
| All |  | T2 | 143 | 11.1 | 6.04 | -4.4 | -5.2 to -3.6 | <0.001 | -0.85 |
| All |  | T3 | 56 | 9.1 | 5.42 | -5.5 | -6.7 to -4.3 | <0.001 | -1.08 |

^a^ Dep: Depression: SAD: Social Anxiety Disorder; PD: Panic Disorder; ^b^ Fixed effect of time adjusted for age, gender and hospital; ^c^ ES = Standardized effect size (Cohen’s *d*)

**Table S2.** Observed and predicted patient-reported outcomes over time adjusted by additional covariates.

|  |  |  | **Regression Coefficient for Time** | | |
| --- | --- | --- | --- | --- | --- |
| **Group** | **Outcome** | **Time point** | **Estimate  ^a^** | **95% CI** | **ES ^b^** |
| Depression  Depression | PHQ-9 | T2 | -4.4 | -5.16 to -3.56 | -0.85 |
|  | PHQ-9 | T3 | -5.5 | -6.64 to -4.28 | -1.07 |
| Social Anxiety  Social Anxiety | SPIN | T2 | -12.8 | -15.57 to -9.96 | -1.08 |
|  | SPIN | T3 | -15.0 | -19.33 to -10.69 | -1.26 |
| Panic Disorder  Panic Disorder | PDSS | T2 | -4.4 | -5.45 to -3.27 | -0.79 |
|  | PDSS | T3 | -5.3 | -6.65 to -3.93 | -0.96 |
| All  All  All  All  All | WSAS | T2 | -4.5 | -5.31 to -3.68 | -0.58 |
|  | WSAS | T3 | -6.6 | -7.75 to -5.42 | -0.85 |
|  | EQ-5D-5L | T2 | 0.10 | 0.08 to 0.13 |  |
|  | EQ-5D-5L | T3 | 0.12 | 0.09 to 0.15 |  |
|  | Sick leave (days) | T2 | -3.3 | -5.56 to -1.06 |  |
|  |  | T3 | -6.1 | -9.65 to -2.47 |  |

^a^ Fixed effect of time, adjusted for age, gender, hospital, referral pathway (self or GP-referred), employment status at baseline, and medication use at baseline, as covariates. ^b^ ES = Standardized effect size (Cohen’s *d*).

**Table S3.** Observed and predicted patient-reported outcomes over time for patients completing treatment (treatment completion is defined as 4 modules completed in the depression group and 5 modules completed in the social anxiety and panic disorder groups; N=294).

|  |  |  | **Regression Coefficient for Time** | | |
| --- | --- | --- | --- | --- | --- |
| **Group** | **Outcome** | **Time point** | **Estimate  ^a^** | **95% CI** | **ES ^b^** |
| Depression | PHQ-9 | T2 | -4,5 | -5,4 to -3,6 | -0,88 |
| Depression | PHQ-9 | T3 | -5,6 | -6,9 to -4,3 | -1,10 |
| Social Anxiety | SPIN | T2 | -14,1 | -18 to -10,3 | -1,19 |
| Social Anxiety | SPIN | T3 | -16,1 | -21,2 to -11 | -1,36 |
| Panic Disorder | PDSS | T2 | -3,9 | -5,1 to -2,7 | -0,71 |
| Panic Disorder | PDSS | T3 | -5,9 | -7,4 to -4,4 | -1,07 |
| All | WSAS | T2 | -4,6 | -5,5 to -3,6 | -0,59 |
| All | WSAS | T3 | -6,5 | -7,8 to -5,2 | -0,84 |
| All | EQ-5D-5L-5L | T2 | 0,10 | 0,1 to 0,1 | 0,44 |
| All | EQ-5D-5L-5L | T3 | 0,12 | 0,1 to 0,2 | 0,50 |
| All | Sick leave (days) | T2 | -5,2 | -7,8 to -2,7 | -0,44 |
| All | Sick leave (days) | T3 | -8,2 | -12,6 to -3,9 | -0,70 |

^a^ Fixed effect of time adjusted for age, gender and hospital; ^b^ ES = Standardized effect size (Cohen’s *d*)

**Table S4.** Multiple imputation of missing data using chained equations (MICE).

|  |  |  | Unimputed (Observed) | | | MICE | | |  |
| --- | --- | --- | --- | --- | --- | --- | --- | --- | --- |
|  |  |  |  | Regression Coefficient for Time | |  | Regression Coefficient for Time | |  |
| Group ^a^ | Outcome | Time | Mean ^b^ | Estimate ^c^ | CI | Mean ^b^ | Estimate ^c^ | 95% CI |  |
| Dep | PHQ9 | T1 | 15.90 |  |  | 15.90 |  |  |  |
| Dep | PHQ9 | T2 | 11.14 | -4.36 | -5.2 to -3.6 | 11.75 | -4.16 | -5.01 to -3.3 | |
| Dep | PHQ9 | T3 | 9.13 | -5.52 | -6.7 to -4.3 | 10.90 | -5.00 | -6.23 to -3.77 | |
| SAD | SPIN | T1 | 40.28 |  |  | 40.32 |  |  | |
| SAD | SPIN | T2 | 26.59 | -13.21 | -16.0 to -10.4 | 27.86 | -12.46 | -16.09 to -8.84 | |
| SAD | SPIN | T3 | 22.39 | -15.48 | -19.9 to -11.1 | 25.14 | -15.19 | -22.85 to -7.52 | |
| PD | PDSS | T1 | 12.32 |  |  | 12.33 |  |  | |
| PD | PDSS | T2 | 7.78 | -4.38 | -5.5 to -3.3 | 7.82 | -4.51 | -5.71 to -3.31 | |
| PD | PDSS | T3 | 6.45 | -5.32 | -6.7 to -4 | 6.64 | -5.69 | -7.08 to -4.31 | |
| All | WSAS | T1 | 20.99 |  |  | 21.00 |  |  | |
| All | WSAS | T2 | 15.80 | -4.63 | -5.4 to -3.8 | 16.52 | -4.48 | -5.44 to -3.52 | |
| All | WSAS | T3 | 12.16 | -6.64 | -7.9 to -5.5 | 15.33 | -5.67 | -7.14 to -4.19 | |
| All | EQ-5D-5L | T1 | 0.59 |  |  | 0.59 |  |  | |
| All | EQ-5D-5L | T2 | 0.72 | 0.11 | 0.08 to 0.13 | 0.70 | 0.11 | 0.09 to 0.14 | |
| All | EQ-5D-5L | T3 | 0.78 | 0.12 | 0.09 to 0.15 | 0.73 | 0.13 | 0.1 to 0.17 | |

^a^ Dep: Depression: SAD: Social Anxiety Disorder; PD: Panic Disorder; ^b^ Descriptive mean; ^c^ Fixed effect of time adjusted for age, gender and hospital.

**Table S5.** Missing not at random scenarios with delta adjustment.

|  |  |  |  |  | **Regression Coefficient for Time** | |
| --- | --- | --- | --- | --- | --- | --- |
|  | **Group ^a^** | **Outcome** | **Time** | **Mean ^b^** | **Estimate ^c^** | **95% CI** |
| Delta adjustment: 10% | |  |  |  |  |  |
|  | Dep | PHQ-9 | T1 | 15.90 |  |  |
|  | Dep | PHQ-9 | T2 | 12.34 | -3.57 | -4.44 to -2.7 |
|  | Dep | PHQ-9 | T3 | 11.09 | -4.81 | -6.05 to -3.57 |
|  | SAD | SPIN | T1 | 40.32 |  |  |
|  | SAD | SPIN | T2 | 29.23 | -11.09 | -14.77 to -7.41 |
|  | SAD | SPIN | T3 | 25.54 | -14.79 | 22.48 to -7.09 |
|  | PD | PDSS | T1 | 12.33 |  |  |
|  | PD | PDSS | T2 | 8.24 | -4.10 | -5.32 to -2.87 |
|  | PD | PDSS | T3 | 6.84 | -5.50 | -6.9 to -4.09 |
|  | All | WSAS | T1 | 21.00 |  |  |
|  | All | WSAS | T2 | 17.35 | -3.64 | -4.62 to -2.67 |
|  | All | WSAS | T3 | 15.60 | -5.40 | -6.88 to -3.91 |
|  | All | EQ-5D-5L | T1 | 0.59 |  |  |
|  | All | EQ-5D-5L | T2 | 0.67 | 0.07 | 0.05 to 0.1 |
|  | All | EQ-5D-5L | T3 | 0.71 | 0.11 | 0.08 to 0.15 |
| Delta adjustment: 20% | |  |  |  |  |  |
|  | Dep | PHQ-9 | T1 | 15.90 |  |  |
|  | Dep | PHQ-9 | T2 | 12.92 | -2.98 | -3.88 to -2.09 |
|  | Dep | PHQ-9 | T3 | 11.28 | -4.62 | -5.88 to -3.37 |
|  | SAD | SPIN | T1 | 40.32 |  |  |
|  | SAD | SPIN | T2 | 30.60 | -9.72 | -13.47 to -5.97 |
|  | SAD | SPIN | T3 | 25.93 | -14.39 | -22.11 to -6.66 |
|  | PD | PDSS | T1 | 12.33 |  |  |
|  | PD | PDSS | T2 | 8.65 | -3.68 | -4.94 to -2.42 |
|  | PD | PDSS | T3 | 7.04 | -5.30 | -6.73 to -3.87 |
|  | All | WSAS | T1 | 21.00 |  |  |
|  | All | WSAS | T2 | 18.19 | -2.81 | -3.81 to -1.81 |
|  | All | WSAS | T3 | 15.87 | -5.12 | -6.63 to -3.62 |
|  | All | EQ-5D-5L | T1 | 0.59 |  |  |
|  | All | EQ-5D-5L | T2 | 0.63 | 0.03 | 0.01 to 0.06 |
|  | All | EQ-5D-5L | T3 | 0.69 | 0.10 | 0.06 to 0.13 |
| Delta adjustment: 30% | |  |  |  |  |  |
|  | Dep | PHQ-9 | T1 | 15.90 |  |  |
|  | Dep | PHQ-9 | T2 | 13.51 | -2.39 | -3.32 to -1.47 |
|  | Dep | PHQ-9 | T3 | 11.47 | -4.44 | -5.71 to -3.16 |
|  | SAD | SPIN | T1 | 40.32 |  |  |
|  | SAD | SPIN | T2 | 31.97 | -8.35 | -12.18 to -4.51 |
|  | SAD | SPIN | T3 | 26.33 | -13.99 | -21.75 to -6.22 |
|  | PD | PDSS | T1 | 12.33 |  |  |
|  | PD | PDSS | T2 | 9.07 | -3.27 | -4.56 to -1.98 |
|  | PD | PDSS | T3 | 7.23 | -5.10 | -6.56 to -3.64 |
|  | All | WSAS | T1 | 21.00 |  |  |
|  | All | WSAS | T2 | 19.03 | -1.97 | -3 to -0.94 |
|  | All | WSAS | T3 | 16.15 | -4.85 | -6.38 to -3.33 |
|  | All | EQ-5D-5L | T1 | 0.59 |  |  |
|  | All | EQ-5D-5L | T2 | 0.59 | **-0.01** | -0.03 to 0.02 |
|  | All | EQ-5D-5L | T3 | 0.67 | 0.08 | 0.04 to 0.11 |

^a^ Dep: Depression: SAD: Social Anxiety Disorder; PD: Panic Disorder; ^b^ Descriptive mean; ^c^ Fixed effect of time adjusted for age, gender and hospital

### Patient-Reported Outcomes by Hospital

We supplemented the comparison of outcomes between all hospitals in the main analysis by grouping hospitals depending on specific characteristics. We compared H1 with all the other hospitals since H1 introduced the eCoping treatment in Norway and has the longest experience in offering this treatment. Results presented in Figure S1 show that there was no difference in outcomes between H1 and the other hospitals. We also compared H1 and H2 with H3 and H4, since H1 and H2 are both large university hospitals that allow for self-referral, whereas H3 and H4 are smaller hospitals that do no allow for self-referral by patients. The results presented in Figure S2 show that there was no difference in outcomes between these pairs of hospitals. Finally, we adjusted for additional patients characteristics (i.e. referral pathway, medication use and employment status) in our mixed models and found that this did not alter the outcomes between hospitals (Figure S3).

Likelihood ratio (LR) tests for hospitals´ interaction with time indicated that the interaction was significant for WSAS scores comparing H1 with all other hospitals (p=0.01), and for GAD-7 scores comparing H1 and H2 with H3 and H4 (p=0.02). A closer inspection reveals that the baseline WSAS scores were a mean (95% CI) 1.44 points (-0.1 to 2.99 points) higher at H1, whereas the baseline GAD-7 scores were 1.57 points (2.47 to 0.67 points) higher at H3 and H4 compared to the other hospitals. There was no difference in outcomes at T2 and T3 for the respective measures.

**Figure S1.** Estimated patient-reported outcomes by hospital over time, H1 vs. H2-H4.


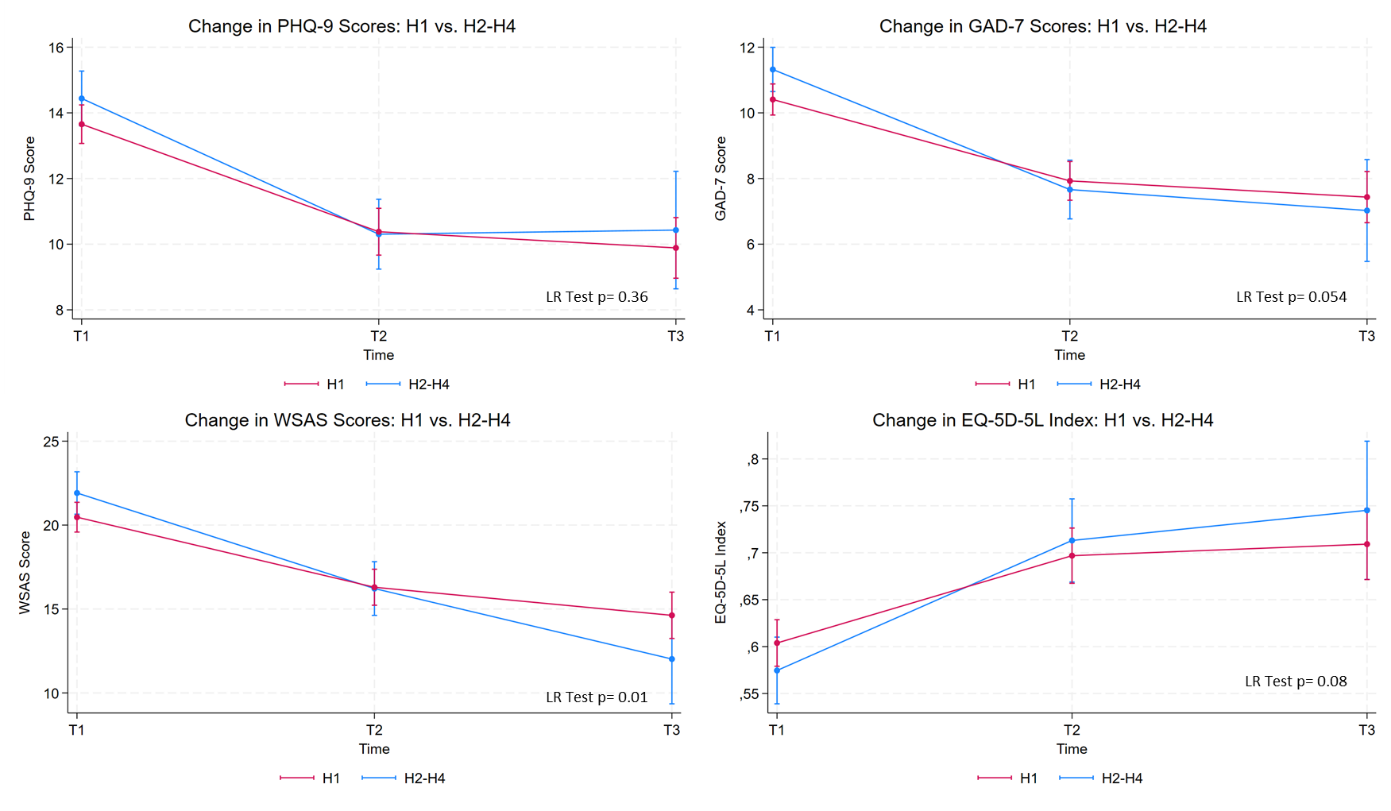


**Figure S2.** Estimated patient-reported outcomes by hospital over time, H1-H2 vs. H3-H4.


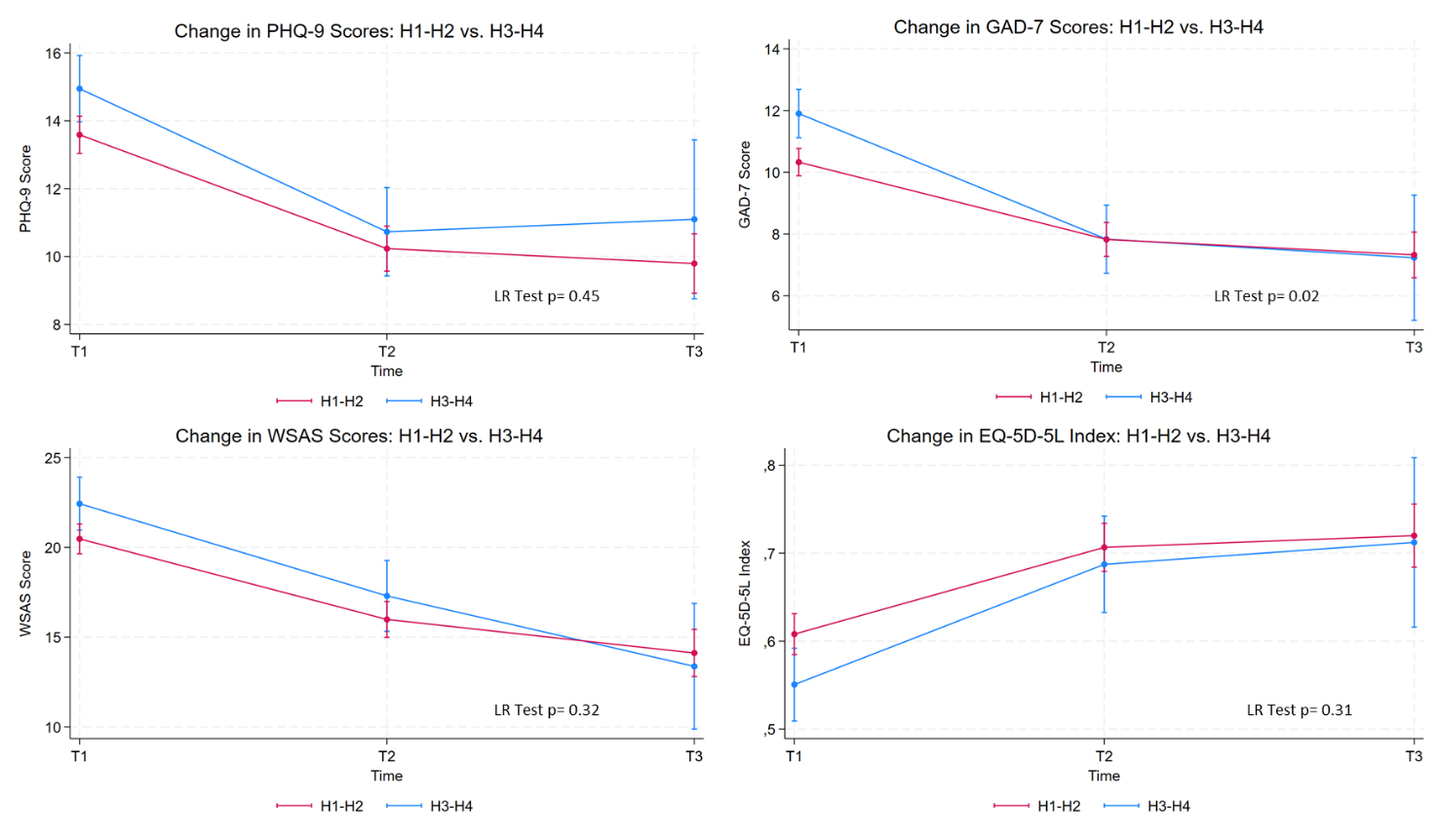


**Figure S3.** Estimated patient-reported outcomes by hospital over time, adjusted for additional covariates.


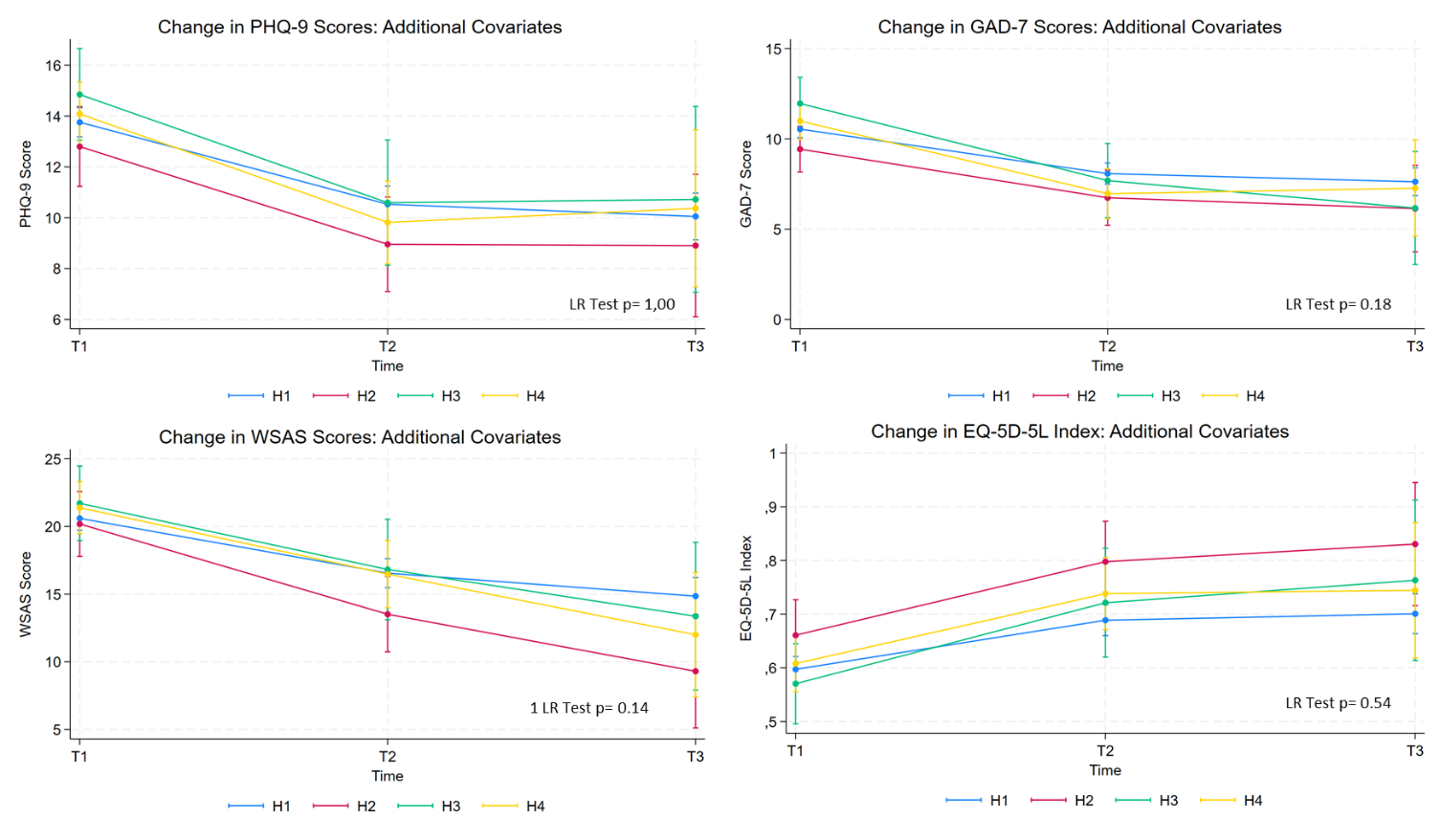


### **Program Costs**

Data collection and data sources: Cost information was collected through close collaboration with participating hospitals. Information on infrastructure costs were collected from legal agreements between the participating hospitals, legal agreements between hospitals and the company Checkware who provided the technical platform, from budgetary documents provided by the participating hospitals and by structured interviews with administrative staff. Only specific iCBT specific technical platform and implementation costs were included, and no broader IT costs like electronic patient journal cost were included. The share of infrastructure costs for the study population was calculated by dividing number of study participants by total number of patients receiving iCBT each year.

Information on therapist costs was in a similar way collected from close contact with clinicians, with administrative staff, from quarterly reports and from questionnaires.

**Table S6. Detailed costs per hospital, in 2022** euros.

| 2022 | | H1 | H2 | H3 | H4 |
| --- | --- | --- | --- | --- | --- |
|  | Total hospital infrastructure cost, per year | 186,406 | 57,944 | 48,073 | 54,311 |
|  | Share of infrastructure costs, study population, per year | 139,392 | 47,163 | 4,370 | - |
|  | Number of patients participating in study, per year | 169 | 35 | 10 | - |
|  | Infrastructure cost per patient | 824.8 | 1,347.5 | 437.0 | - |
|  | - maintenance cost per patient | 137.6 | 593.1 | 333.0 | - |
|  | - implementation costs per patient | 687.2 | 754.5 | 104.0 | - |
|  | Unit cost |  |  |  |  |
|  | - Therapist cost per hour | 51.9 | 50.0 | 54.2 | - |
|  |  |  |  |  |  |
| 2023 | | H1 | H2 | H3 | H4 |
|  | Total hospital infrastructure cost, per year | 186,274 | 56,229 | 53,751 | 50,043 |
|  | Share of infrastructure costs, study population, per year | 135,770 | 45,519 | 12,343 | 35,852 |
|  | Number of patients participating in study, per year | 207 | 17 | 31 | 96 |
|  | Infrastructure cost per patient | 655.9 | 2,677.6 | 398.2 | 373.5 |
|  | - maintenance cost per patient | 103.6 | 1,159.2 | 234.0 | 208.2 |
|  | - implementation costs per patient | 552.3 | 1,518.4 | 164.2 | 165.3 |
|  | Unit cost |  |  |  |  |
|  | - Therapist cost per hour | 53.9 | 48.7 | 55.0 | 51.8 |

### STATA Code

| **Variable Name** | **Description** |
| --- | --- |
| phq9 | PHQ-9 |
| wsas | WSAS |
| gad7 | GAD-7 |
| eq5d | EQ-5D-5L |
| spin | SPIN |
| pdss | PDSS |
| sickdays | Sick days |
| Age | Age in years |
| Gender | Male, Female |
| Hospital | H1 to H4 |
| ParticipantID | Participant ID |
| Treatment | Treatment group |
| T1_EmploymentStatus | Baseline employment status |
| T1_MedicationUse | Baseline medication use |
| ReferralType | GP or self-referred |
| SumModules | Number of modules completed |
| time | T1 to T3 |
| Suffix _1 to _3 | Time points T1 to T3 |

*** Example Code: Fixed effect of time (Main Analysis) ***

mixed phq9 Age Gender Hospital if Treatment == 1 || ParticipantID: // (Treatment 1 is Depression)

mixed spin Age Gender Hospital if Treatment == 2 || ParticipantID: // (Treatment 2 is Social Anxiety)

mixed pdss Age Gender Hospital if Treatment == 3 || ParticipantID: // (Treatment 3 is Panic Disorder)

mixed wsas Age Gender Hospital || ParticipantID:

mixed eq5d Age Gender Hospital || ParticipantID:

mixed sickdays Age Gender Hospital || ParticipantID:

*** Example Code: Hospital level effect (Main Analysis) ***

foreach outcome in phq9 gad7 wsas eq5d5l {

* Fit model without interaction

mixed `outcome' ib1.time i.Gender Age ib1.Hospital || ParticipantID:, stddeviations

estimates store A_`outcome'

* Fit model with hospital*time interaction

mixed `outcome' ib1.time i.Gender Age i.Hospital#i.time || ParticipantID:, stddeviations

estimates store B_`outcome'

* Likelihood ratio test

lrtest B_`outcome' A_`outcome'

}

*** Multiple Imputation Chained Equations (MICE) ***

mi set wide

mi register imputed ///

phq9_1 phq9_2 phq9_3 ///

gad7_1 gad7_2 gad7_3 ///

eq5d_1 eq5d_2 eq5d_3 ///

spin_1 spin_2 spin_3 ///

pdss_1 pdss_2 pdss_3 ///

wsas_1 wsas_2 wsas_3 ///

Age SumModules ///

T1_EmploymentStatus T1_MedicationUse ReferralType

mi register regular Gender ParticipantID Hospital Treatment

mi impute chained ///

(pmm, knn(5)) phq9_1 ///

(pmm, knn(5)) phq9_2 ///

(pmm, knn(5)) phq9_3 ///

(pmm, knn(5)) gad7_1 ///

(pmm, knn(5)) gad7_2 ///

(pmm, knn(5)) gad7_3 ///

(pmm, knn(5)) eq5d_1 ///

(pmm, knn(5)) eq5d_2 ///

(pmm, knn(5)) eq5d_3 ///

(pmm, knn(5)) spin_1 ///

(pmm, knn(5)) spin_2 ///

(pmm, knn(5)) spin_3 ///

(pmm, knn(5)) pdss_1 ///

(pmm, knn(5)) pdss_2 ///

(pmm, knn(5)) pdss_3 ///

(pmm, knn(5)) wsas_1 ///

(pmm, knn(5)) wsas_2 ///

(pmm, knn(5)) wsas_3 ///

Age ///

(pmm, knn(5)) SumModules ///

(pmm, knn(5)) T1_EmploymentStatus ///

(pmm, knn(5)) T1_MedicationUse ///

(pmm, knn(5)) ReferralType ///

= Gender ParticipantID Hospital Treatment, ///

add(100) rseed(12345)

mi reshape long phq9_ gad7_ eq5d_ spin_ pdss_ wsas_, i(ParticipantID) j(time)

*** Missing Not At Random: Delta Adjustment ***

// Delta adjustment: 10%

foreach var in phq9 gad7 spin pdss wsas {

replace `var' = `var' * 1.10 if _mi_m > 0 & inlist(time, 2, 3)

}

replace eq5d = eq5d * 0.90 if _mi_m > 0 & inlist(time, 2, 3)

// Delta adjustment: 20%

foreach var in phq9 gad7 spin pdss wsas {

replace `var' = `var' * 1.20 if _mi_m > 0 & inlist(time, 2, 3)

}

replace eq5d = eq5d * 0.80 if _mi_m > 0 & inlist(time, 2, 3)

// Delta adjustment: 30%

foreach var in phq9 gad7 spin pdss wsas {

replace `var' = `var' * 1.30 if _mi_m > 0 & inlist(time, 2, 3)

}

replace eq5d = eq5d * 0.70 if _mi_m > 0 & inlist(time, 2, 3)

### Abbreviations

CI Confidence Interval

Dep Depression

EQ-5D EuroQol 5D-5L

GAD-7 Generalized Anxiety Disorder Scale 7

iCBT Internet-delivered cognitive behavioral therapy

ICER Incremental cost-effectiveness ratio

ITT Intention to Treat

MAR Missing At Random

MICE Multiple Imputation Chained Equations

MNAR Missing Not At Random

PD Panic Disorder

PDSS Panic Disorder Severity Scale

PHQ-9 Patient Health Questionnaire 9

RCT Randomized Clinical Trial

REK Norwegian Regional Ethics Committee

SAD Social Anxiety Disorder

SD Standard deviation

SPIN Social Phobia Inventory

WSAS Work and Social Adjustment Scale
